# Seasonal Shifts in Bacterial Community Structures in the Lateral Root of Sugar Beet Grown in an Andosol Field in Japan

**DOI:** 10.1264/jsme2.ME22071

**Published:** 2023-02-09

**Authors:** Seishi Ikeda, Kazuyuki Okazaki, Hiroyuki Takahashi, Hirohito Tsurumaru, Kiwamu Minamisawa

**Affiliations:** 1 Memuro Research Station, Hokkaido Agricultural Research Center, National Agriculture and Food Research Organization, 9–4 Shinsei-minami, Memuro, Kasai-gun, Hokkaido 082–0081, Japan; 2 Faculty of Agriculture, Kagoshima University, 1–21–24, Korimoto, Kagoshima 890–0065, Japan; 3 Graduate School of Life Science, Tohoku University, 2–1–1 Katahira, Aoba-ku, Sendai, Miyagi 980–8577, Japan

**Keywords:** bacterial community ana­lysis, lateral root, plant growth-promoting bacteria, seasonal shifts, sugar beet

## Abstract

To investigate functional plant growth-promoting rhizobacteria in sugar beet, seasonal shifts in bacterial community structures in the lateral roots of sugar beet were examined using amplicon sequencing ana­lyses of the 16S rRNA gene. Shannon and Simpson indexes significantly increased between June and July, but did not significantly differ between July and subsequent months (August and September). A weighted UniFrac principal coordinate ana­lysis grouped bacterial samples into four clusters along with PC1 (43.8%), corresponding to the four sampling months in the order of sampling dates. Taxonomic ana­lyses revealed that bacterial diversity in the lateral roots was exclusively dominated by three phyla (*Actinobacteria*, *Bacteroidetes*, and *Proteobacteria*) in all samples examined. At the lower taxonomic levels, the dominant taxa were roughly classified into three groups. Therefore, the relative abundances of seven dominant genera (*Janthinobacterium*, *Kribbella*, *Pedobacter*, *Rhodanobacter*, *Sphingobium*, *Sphingopyxis*, and *Streptomyces*) were the highest in June and gradually decreased as sugar beet grew. The relative abundances of eight taxa (*Bradyrhizobiaceae*, *Caulobacteraceae*, *Chitinophagaceae*, *Novosphingobium*, *Phyllobacteriaceae*, *Pseudomonas*, *Rhizobiaceae*, and *Sphingomonas*) were mainly high in July and/or August. The relative abundances of six taxa (unclassified *Comamonadaceae*, *Cytophagaceae*, unclassified *Gammaproteobacteria*, *Haliangiaceae*, unclassified *Myxococcales*, and *Sinobacteraceae*) were the highest in September. Among the dominant taxa, 12 genera (*Amycolatopsis*, *Bradyrhizobium*, *Caulobacter*, *Devosia*, *Flavobacterium*, *Janthinobacterium*, *Kribbella*, *Kutzneria*, *Pedobacter*, *Rhizobium*, *Rhodanobacter*, and *Steroidobacter*) were considered to be candidate groups of plant growth-promoting bacteria based on their previously reported beneficial traits as biopesticides and/or biofertilizers.

Approximately 20% of the world’s sucrose production is derived from sugar beet (*Beta vulgaris* L.), which is the most important crop in temperate regions for sugar industry (Godshall, 2012). Sugar beet has been attracting increasing interest as a source of bioenergy (Koga, 2008) because of its higher biomass production than other temperate crops under harsh environmental conditions (Steingrobe, 2001, 2005; de Vries *et al.*, 2010). However, the mechanisms underlying the high productivity and tolerance of sugar beet against diverse biotic and abiotic stresses have not yet been elucidated in detail. One possible explanation for these features of sugar beet may be colonization by plant growth-promoting bacteria (PGPB), which confer stress tolerance and growth-promoting effects on crops (Steingrobe, 2005; Toyota and Watanabe, 2013). Therefore, a more detailed understanding of the diversity and functionality of plant-associated microbes is considered to be a very important key step in the construction of a sustainable agricultural system that takes advantage of the beneficial functionalities of PGPB as a green technology (Bulgari *et al.*, 2019).

Previous studies reported diverse species of bacteria as PGPB in sugar beet (Kloepper *et al.*, 1980; Dunne *et al.*, 1998; Çakmakçi *et al.*, 1999, 2001, 2006; Shi *et al.*, 2009; Okazaki *et al.*, 2021). However, difficulties are associated with the practical utilization of PGPB (Raymaekers *et al.*, 2020); the use of PGPB as a biopesticide or biofertilizer often fails to produce stable growth-promoting effects under field conditions. One possible explanation for this failure is the insufficient consideration of the colonization ability of PGPB on a plant tissue under field conditions (Lugtenberg and Kamilova, 2009; Compant *et al.*, 2010). Microbial community ana­lyses recently revealed that the relative abundance of plant-associated bacteria was affected by a number of environmental factors, such as the plant growth stage. and agronomical practices, including the application of fertilizers and pesticides, in diverse plant species (Ikeda *et al.*, 2014; Unno *et al.*, 2015; Masuda *et al.*, 2016; Hara *et al.*, 2019). Under these environmental factors, the stable colonization of PGPB on and/or in plant tissues during crop cultivation needs to be regarded as a key characteristic of PGPB for their practical use in agriculture. It is also reasonable to consider the colonization ability of a plant-associated microorganism to be reflected by its abundance in a plant tissue, as demonstrated by mole­cular community ana­lyses in conjunction with current environmental DNA sequencing technologies. However, the impact of plant growth stages on the microbial community of an underground plant tissue has not been widely examined in a time series ana­lysis under field conditions due to the associated high costs and labor demands.

In the case of sugar beet, underground plant tissues are anatomically divided into two types of tissues: the taproot and lateral roots. While the taproot is structurally durable and mainly contributes to the uptake of water from deep soil layers as well as the accumulation of sugar, the lateral roots are fragile and involved in the uptake of nutrients and water from the surface soil layer. Therefore, the lateral roots are regarded as an active site for PGPB to enhance nutrient uptake from soil to sugar beet. Okazaki *et al.* (2021) successfully isolated several novel PGPB from the lateral roots of sugar beet. Although a bacterial community ana­lysis of the lateral roots has been conducted, it did not provide many ecological aspects for PGPB due to the use of a classical clone library ana­lysis without a sufficient amount of sequence data at one time point during the cultivation of sugar beet. Similarly, the reported persistency of other PGPB in the tissues of sugar beet was not investigated under field conditions.

Regarding the rhizosphere of sugar beet, microbial community ana­lyses with recent mole­cular biological technologies have targeted rhizosphere soil (Mendes *et al.*, 2011; Zachow *et al.*, 2014; Hudz and Skivka, 2021) and the taproot (Shi *et al.*, 2014; Tsurumaru *et al.*, 2015), but not the lateral roots, except in our study described above (Okazaki *et al.*, 2021). A microbial community ana­lysis using an automated ribosomal intergenic spacer previously demonstrated that plant growth stages influenced the diversity of the rhizosphere microbial community in tuber peelings of sugar beet (Houlden *et al.*, 2008). Similarly, the growth stage of sugar beet is expected to have an impact on the microbial community of lateral roots. Based on these findings, we herein investigated the impact of sugar beet growth stages (late seedling, vigorous growth, and sugar accumulation) on bacterial community structures in lateral roots using high-throughput sequencing to identify dominant bacterial groups at each growth stage and also understand the dynamics of these groups under field conditions for the practical and efficient use of PGPB in agriculture.

## Materials and Methods

### Plant materials and soil sampling

Seeds of the sugar beet cultivar “Rycka” were sown in pots (paper pot no. 1; Nippon Beet Sugar Manufacturing) filled with field soil supplemented with chemical fertilizer and soil conditioner (Matsuhira *et al.*, 2022) under greenhouse conditions on March 13, 2014. The seedlings of sugar beet were planted in a plot (eight rows with a row length of 6.75 m) of an Andosol experimental field at the Hokkaido Agricultural Research Center (Memuro, Hokkaido, Japan, 42°89′20″N, 143°07′70″E, 94‍ ‍m‍ ‍a.s.l.) on April 22, 2014. Inter- and intra-row distances were 60 and 22.5‍ ‍cm, respectively. The field was dressed with the commercial fertilizer S014 (150, 315, and 210‍ ‍kg ha^–1^ for N, P_2_O_5_, and K_2_O, respectively; Hokuren Fertilizer) as basal fertilization. Based on a visual inspection, six healthy plants were randomly sampled from the plot every month between June and September (June 16, July 15, August 12, and September 17, 2014). At the experimental site, sampling dates roughly corresponded to the late seedling (June), vigorous growth (July and August), and sugar accumulation (September) stages (Fig. S1). The roots of individual plants were carefully washed with tap water to remove loosely adhering soil and organic debris and were then rinsed with sterilized water. The lateral roots on the taproot were individually collected using a sterilized forceps. Six lateral root samples per month were stored at –30°C until used for DNA preparation. Soil samples were collected from five sampling sites in the plot by an auger (between a depth of 5 and 15‍ ‍cm) after the removal of surface soil on July 15, 2014 and were combined as a composite soil sample. The chemical characteristics of the soil sample were assessed by the Tokachi Nokyoren Agricultural Research Institute (Obihiro, Hokkaido, Japan) as described elsewhere (Okada and Harada, 2007) (Table S1).

### Sequencing and editing of sequence data prior to statistical analyses

The lateral roots of an individual plant (approximately 1 g) were grounded in liquid nitrogen with a mortar and pestle. A portion of the pulverized sample (0.4 g) was transferred into a Lysing Matrix E tube (MP Biomedicals), and a DNA sample was prepared as previously described (Ikeda *et al.*, 2004), except that a homogenizer (FastPrep^®^24, MP Biomedicals) was used for the bead beating step (30‍ ‍s at 5.5 ms^–1^ and room temperature) and a DEAE-cellulose column treatment was omitted. Pelleted DNA was then washed with 85% ethanol and suspended in 100‍ ‍μL of TE buffer (pH 7.6).

In the bacterial community ana­lysis, PCR amplification for the bacterial 16S rRNA gene was conducted using the 1st-515f_MIX and 1st-806r_MIX primer set that amplified the V3–V4 region of the bacterial 16S rRNA gene (Caporaso *et al.*, 2011). PCR amplification was performed as follows: 94°C for 2‍ ‍min, 30 cycles of a program (94°C for 30‍ ‍s, 50°C for 30‍ ‍s, and 72°C for 30 s), and then final extension at 72°C for 5‍ ‍min. PCR amplicons were used as template DNA for second PCR and the paired-end sequence (2×300 bp) on a MiSeq sequencer using MiSeq Reagent Kit v3 (Illumina) as described by Nakamura *et al.* (2020).

The extraction of raw reads containing a perfect primer sequence, the removal of the primer and 50 bases of the 3′end sequences, and trimming (minimum length of 40 bases; minimum average quality score of 20) were performed as described by Nakamura *et al.* (2020). Trimmed reads were merged (minimum overlap 10 bases; average merged length 250 bases; average length of individual reads 230 bases) using FLASH software (Magoc and Salzberg, 2011). Taxonomic assignment for the merged sequences was performed using a script of Qiime (Caporaso *et al.*, 2010), and sequences classified into archaea, chloroplasts, mitochondria, and unassigned sequences at the domain level were removed. The removal of chimera and noise sequences, the generation of amplicon sequence variants (ASVs), and their representative sequences were conducted using the data2 Qiime2 plugin with default settings (Bolyen *et al.*, 2019). After rarefaction to 5,491 reads per sample, taxonomic assignment for the representative sequences was performed with the feature-classifier Qiime2 plugin by comparisons with 97% OTUs of Greengenes, and alpha-diversity measures and UniFrac principal coordinate ana­lysis (PCoA) were conducted with the script of Qiime.

### Statistical ana­lysis

A one-way ANOVA and Tukey’s HSD (honestly significant difference) test were performed using JMP software version 12 (SAS Institute) for multiple comparisons of the relative abundances of bacterial taxa among sampling months.

### Nucleotide sequence accession numbers.

The raw reads used in the present study were deposited into the NCBI SRA database (BioProject accession number: PRJNA826328).

## Results

### Alpha-diversity measures and UniFrac PCoA

Alpha-diversity measures with the relative abundance data of ASVs based on 5,491 reads per sample revealed that all diversity indexes examined for the lateral root-associated bacteria (LR bacteria) of sugar beet significantly increased between June and July or August (Table 1). The numbers of singleton, ASV, Chao1, and ACE significantly increased between June and August and no significant differences were observed between August and September. Shannon and Simpson indexes significantly increased between June and July, but did not significantly differ between July and subsequent months (August and September).

**Fig. 1.**
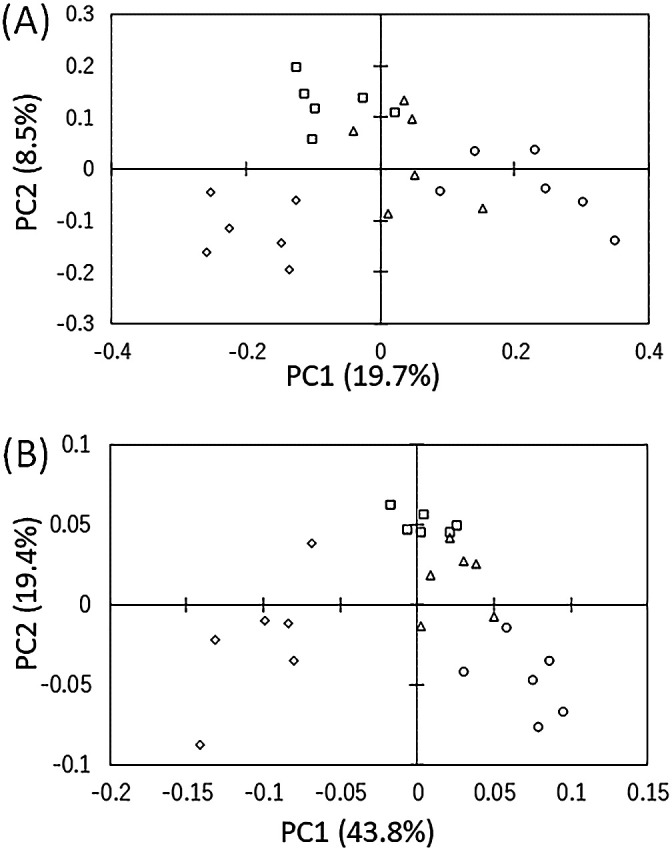
Principal coordinate ana­lysis of partial sequences of 16S ribosomal RNA gene amplicon libraries (amplicon sequence variants, ASVs) for lateral root-associated bacteria of sugar beet in different sampling months. The ordination was constructed using unweighted (A) and weighted (B) UniFrac distances by relative abundances. Each symbol represents the phylogenetic composition of lateral root-associated bacteria in different sampling months. Sampling months: circle (○): June; triangle (△): July; Square (□): August; diamond (◇): September.

The results of unweighted and weighted UniFrac PCoA showed that bacterial samples were mainly grouped into four clusters along with PC1 (19.7 and 43.8%, respectively), corresponding to the four sampling months in the order of the sampling time (between June and September) (Fig. 1). PCoA results also indicated shifts in July and August samples along with PC2 (8.5 and 19.4%, respectively) relative to June and September samples. June, July, and August samples were more tightly clustered together in weighted UniFrac PCoA than in unweighted UniFrac PCoA. June and September samples in UniFrac PCoA were more loosely clustered than July and August samples.

### Analyses of taxonomic compositions of and seasonal shifts in bacterial communities in lateral roots of sugar beet

**Fig. 2.**
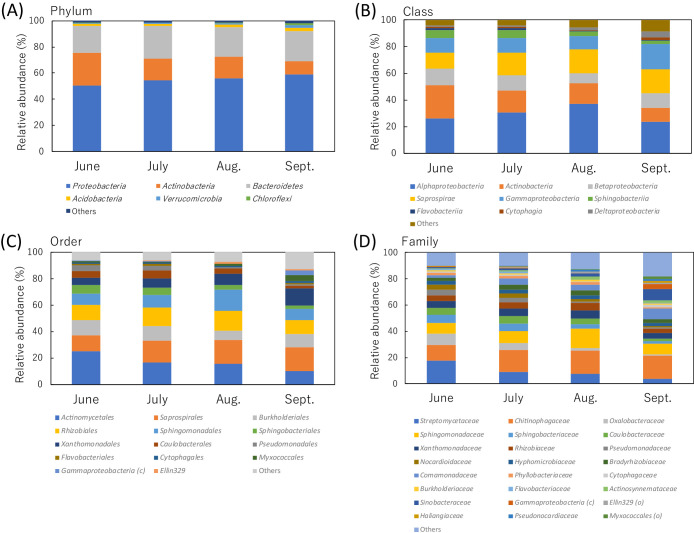
Phylogenetic compositions of lateral root-associated bacteria of sugar beet in different sampling months. The dominant taxa with relative abundances of 1% or more than 1% in any one of the sampling months at the phylum (A), class (B), order (C), and family (D) levels are shown.

The ana­lysis of the taxonomic compositions of all combined sequence data in the present study identified 32 phyla, 85 classes, 174 orders, 295 families, 473 genera, and 542 species. A clustering ana­lysis of sequence data with 100% identity generated 2,734 ASVs. Among these taxa and ASVs, 6 phyla, 9 classes, 14 orders, 24 families, 40 genera, 40 species, and 39 ASVs were identified as dominant taxa or ASVs with relative abundances of 1% or more than 1% in any one of the sampling months. At the phylum level, the relative abundances of three dominant phyla (*Actinobacteria*, *Bacteroidetes*, and *Proteobacteria*) accounted for more than‍ ‍90% (92.2 to 96.1%) in all sampling months (Fig. 2A and Table S2). At the class and order levels, the relative abundances of six dominant classes (*Actinobacteria*, *Alphaproteobacteria*, *Betaproteobacteria*, *Gammaproteobacteria*, *Saprospirae*, and *Sphingobacteriia*) and eight dominant orders (*Actinomycetales*, *Burkholderiales*, *Caulobacterales*, *Rhizobiales*, *Saprospirales*, *Sphingobacteriales*, *Sphingomonadales*, and *Xanthomonadales*) accounted for more than 80% (84.3 to 92.6%) and 70% (74.8 to 85.9%), respectively, in all sampling months (Fig. 2B, C, and Table S2).

The taxonomic compositions of the dominant bacterial groups at the lower taxonomic levels from family to ASV markedly differed among sampling months. At the family level, eleven dominant families (*Bradyrhizobiaceae*, *Caulobacteraceae*, *Chitinophagaceae*, *Comamonadaceae*, *Hyphomicrobiaceae*, *Nocardioidaceae*, *Rhizobiaceae*, *Sphingomonadaceae*, *Sphingobacteriaceae*, *Streptomycetaceae*, and *Xanthomonadaceae*) were present in all sampling months (Fig. 2D). Their relative abundances accounted for more than 70% in June, July, and August (70.2, 71.7, and 73.1%, respectively), but less than 60% in September (55.9%) (Table S2). At the genus level, only eleven dominant genera‍ ‍(*Caulobacter*, *Chitinophaga*, *Devosia*, *Kribbella*, *Niastella*, *Novosphingobium*, *Rhizobium*, *Sphingomonas*, *Streptomyces*, unclassified *Chitinophagaceae*, and unclassified *Comamonadaceae*) were present in all sampling months and their relative abundances accounted for less than 50% (37.3 to 44.8%) in all sampling months (Table S3). The taxonomic compositions of eleven dominant groups at the species level corresponded to those of eleven dominant groups at the genus level as described above, whereas only two groups were taxonomically resolved at the species level (*Caulobacter henricii* and *Chitinophaga arvensicola*) in the Greengenes database. At the ASV level, only five ASVs (ASV_001 [*Streptomyces*], ASV_002 [*Chitinophagaceae*], ASV_003 [*C. henricii*], ASV_005 [*C. arvensicola*], and ASV_006 [*Rhizobium*]) were present as dominant ASVs in all sampling months and their relative abundances accounted for only 22.1, 16.9, 16.3, and 7.8% in June, July, August, and September, respectively (Table 2).

Statistical ana­lyses revealed that the relative abundances of 11 phyla, 32 classes, 52 orders, 84 families, 120 genera, 137 species, and 263 ASVs significantly differed among the sampling months. Among these taxa and ASVs, 4 phyla, 9‍ ‍classes, 14 orders, 21 families, 33 genera, 34 species, and 34 ASVs were identified as dominant taxa or ASVs with relative abundances of 1% or more than 1% in any of the sampling months. At the phylum level, the relative abundances of three phyla (*Chloroflexi*, *Proteobacteria*, and *Verrucomicrobia*) increased between June and September, while that of *Actinobacteria* markedly decreased between June to September (Table 3). Among the dominant phyla, the relative abundances of *Bacteroidetes* and *Acidobacteria* did not significantly differ among the sampling months (Table S2). At the class level, the relative abundances of the dominant classes belonging to *Proteobacteria* and *Actinobacteria* significantly differed among the sampling months, while those belonging to *Bacteroidetes* (*Cytophagia*, *Flavobacteriia*, *Saprospirae*, and *Sphingobacteriia*) did not (Table S2). In *Proteobacteria*, the relative abundances of *Alpha*- and *Betaproteobacteria* were the highest and lowest, respectively, in August. The relative abundances of *Gamma*- and *Deltaproteobacteria* were the highest in September. At the order level, the relative abundances of all dominant orders (Fig. 2C and Table S2) significantly differed among the sampling months (Table 3). The relative abundances of three orders in *Alphaproteobacteria* (*Caulobacterales*, *Rhizobiales*, and *Sphingomonadales*) were slightly higher in July and/or August, while that of *Ellin329* was high in August and September. The relative abundances of orders in *Gamma-*, and *Deltaproteobacteria* were high in September, except for *Pseudomonadales*, the abundance of which was high in June and July. In *Bacteroidetes*, the relative abundances of *Sphingobacteriales* and *Flavobacteriales* were both high in June and July, while that *Cytophagales* was significantly high in September. The relative abundances of *Burkholderiales* and *Saprospirales* remained steady between June and September, but were significantly low in August and June, respectively.

Consistent with the results shown in Table 1 and Fig. 1, statistical ana­lyses of changes in relative abundance at the level from family to ASV revealed a marked shift in community structures and an increase in diversity occurred between June and September at low taxonomic levels, mainly in *Actinobacteria*, *Bacteroidetes*, and *Proteobacteria* (Table 4). Therefore, the relative abundances of dominant taxa in June significantly decreased in September, while those of a number of minor taxa (relative abundance less than 1) in June significantly increased in September as dominant taxa. This was clearly observed at the ASV level (Table 2).

At the family to species levels (Table 4), the relative abundances of seven dominant taxa in June (*C. henricii*, *Janthinobacterium*, *Kribbella*, *Pedobacter*, *Rhodanobacter*, *Sphingobium*, and *Streptomyces*) were the highest and then gradually decreased in subsequent months. The relative abundances of 12 other dominant taxa in June (*Asticcacaulis biprosthecium*, Unclassified *Bradyrhizobiaceae*, unclassified *Chitinophagaceae* [classified as “*Chitinophagaceae*;g__” in‍ ‍the Greengenes database], *C. arvensicola*, *Devosia*, *Mesorhizobium*, *Novosphingobium*, unclassified *Oxalobacteraceae*, *Pseudomonas*, *Rhizobium*, *Sphingomonas*, and *Sphingopyxis*) were higher in July and/or August. Among the minor taxa in June, the relative abundances of eight taxa (*Agrobacterium*, *Amycolatopsis*, *Bradyrhizobium*, *Dokdonella*, unclassified *Ellin329*, *Flavobacterium*, *Kutzneria*, and *Sphingomonas azotifigens*) were higher in July and/or August as dominant taxa. In contrast, the relative abundances of ten minor taxa in June (*Amycolatopsis*, *Bradyrhizobium*, unclassified *Chitinophagaceae* [classified as “*Chitinophagaceae*;__” in the Greengenes database], unclassified *Comamonadaceae*, *Dokdonella*, unclassified *Gammaproteobacteria*, *Haliangiaceae*, unclassified *Myxococcales*, unclassified *Sinobacteraceae*, and *Steroidobacter*) were higher in September as dominant taxa. The relative abundances of only two dominant taxa in June (unclassified *Comamonadaceae* and *Cytophagaceae*) were significantly higher in September. In contrast to the taxa described above, the relative abundances of ten dominant taxa (*Burkholderia*, Unclassified *Cytophagaceae*, Unclassified *Haliangiaceae*, *Hyphomicrobiaceae*, *Lysobacter*, *Niastella*, Unclassified *Rhizobiaceae*, Unclassified *Rhodanobacter*, and Unclassified *Sphingobacteriaceae*), which were 1% or more than 1% in any one of the sampling months, did not significantly differ among sampling months (Table S2 and S3).

At the ASV level, the relative abundances of 12 dominant ASVs in June (ASV_001 [*Streptomyces*], ASV_003 [*C. henricii*], ASV_012 [*Kribbella*], ASV_014 [*Pedobacter*], ASV_015 [*Sphingobium*], ASV_019 [*Rhodanobacter*], ASV_022 [*Oxalobacteraceae*], ASV_030 [*Janthinobacterium*], ASV_032 [*A. biprosthecium*], ASV_038 [*Kribbella*], ASV_039 [*Streptomyces*], and ASV_064 [*Streptomycetaceae*]) decreased in the later months and were all the lowest in September (Table 2). The relative abundances of five dominant ASVs in June (ASV_002 [*Chitinophagaceae*], ASV_005 [*C. arvensicola*], ASV_006 [*Rhizobium*], ASV_007 [*Sphingomonas*], and ASV_020 [*Pseudomonas*]) were the highest in July and/or August and the lowest or low in September. The relative abundances of 17‍ ‍minor ASVs in June were dominant ASVs in the later months, while 12 (ASV_004 [*Bradyrhizobium*], ASV_009 [*Agrobacterium*], ASV_010 [*Novosphingobium*], ASV_011 [*Steroidobacter*], ASV_017 [*Amycolatopsis*], ASV_021 [*Chitinophaga*], ASV_024 [*Sinobacteraceae*], ASV_025 [*Comamonadaceae*], ASV_026 [*Steroidobacter*], ASV_040 [*Comamonadaceae*], ASV_046 [*Gammaproteobacteria*], and ASV_054 [*Gammaproteobacteria*]) were the highest or‍ ‍high in September. The relative abundances of the remaining five minor ASVs in June (ASV_013 [*Novosphingobium*], ASV_016 [*S. azotifigens*], ASV_023 [*Novosphingobium*], ASV_027 [*Dokdonella*], ASV_034 [*Kutzneria*], and ASV_046 [*Gammaproteobacteria*]) were the highest in July and/or August. In contrast to the taxa described above, the relative abundances of four dominant ASVs, which were 1% or more than 1% in any one of the sampling months (ASV_008 [*Niastella*], ASV_018 [*Devosia*], ASV_031 [*Sphingobacteriaceae*], and ASV_033 [*Niastella*]), did not significantly differ among the sampling months (Table 2).

An ana­lysis of taxonomic compositions at the ASV level revealed marked differences among taxa for the level of diversity within a taxon. Seven dominant taxa with relative abundances of 1% or more than 1% in any one of the sampling months (*Amycolatopsis*, *A. biprosthecium*, *C. arvensicola*, *Kutzneria*, and *S. azotifigens*) were represented by one ASV (Table S4). Although the numbers of ASVs for‍ ‍most of the dominant taxa with relative abundances of 1% or more than 1% in any one of the sampling months were less than 20, those for eight taxa showing high abundances in September (*Chitinophagaceae*, unclassified *Comamonadaceae*, *Cytophagaceae*, unclassified *Ellin329*, unclassified *Gammaproteobacteria*, *Haliangiaceae*, unclassified *Myxococcales*, and *Sinobacteraceae*) were 170, 24, 56, 23, 32, 33, 94, and 39, respectively, suggesting high genetic diversity within these taxa (Table S4).

## Discussion

### Alpha-diversity measures and UniFrac PCoA

Regarding the diversity indexes in Table 1, a singleton indicates the number of a unique sequence in a sample (the number of ASVs containing only one read). Chao1 and ACE are estimators for the lower limit of the expected numbers of ASVs based on a theoretically extended sufficiently large data set employing slightly different weighting methods. Shannon and Simpson indexes are diversity indexes that are calculated based on an experimentally obtained data set employing slightly different weighting methods. Based on overall changes in these alpha-diversity measurements, the diversity of LR bacteria increased between June and August and was saturated in August and September (Table 1). Slight differences between the numbers of ASVs and those of Chao1 and ACE suggested that the amount of sequence data in the present study was technically sufficient to evaluate the diversity of LR bacteria. Marked differences in all diversity indexes among sampling months indicated the importance of a time series ana­lysis for evaluating the diversity of LR bacteria of sugar beet. Shannon and Simpson indexes revealed that the abundance of the dominant groups rapidly changed between June and July, while other indexes confirmed a gradual increase in the taxonomic composition mainly in the minor groups between June and August.

The unweighted UniFrac distance represents qualitative differences in microbial community structures among samples based on the presence/absence of data on each of the microbial taxa observed, while the weighted UniFrac distance represents quantitative differences in those based on both of the presence/absence of data on microbial taxa and the abundance of data on each of these taxa (Wu *et al.*, 2010). Unweighted and weighted UniFrac PCoA (Fig. 1) revealed that the continuous succession of bacterial communities during the growth of sugar beet had occurred in the lateral roots. Houlden *et al.* (2008) reported distinct differences in microbial community structures in the rhizosphere (tuber peelings) among the different growth stages of sugar beet. Shifts in clusters in July and August samples to the same direction along with PC2 in unweighted and weighted UniFrac PCoA (8.5 and 19.4% in Fig. 1A and B, respectively) indicated that the bacterial community structures in July and August samples were more similar to each other than to those in June and September samples. The loose cluster of June samples may reflect the small influence of the root on the rhizosphere microbiome due to the low daily minimum temperature and precipitation in June at the experimental site (Fig. S2). The large loose cluster of September samples (Fig. 1B) most likely reflects the senescence of rhizosphere-related organic matter because sugar beet gradually stops vegetative growth in September at the experimental site (Fig. S1) and begins to accumulate sugar from above-ground tissues to the taproot. These results revealed that the bacterial community structure in lateral roots rapidly, markedly, and continuously changed during the growth period of sugar beet, indicating the importance of time series ana­lyses to evaluate bacterial diversity in the rhizosphere.

### Overview of taxonomic features of lateral root-associated bacteria

Although sugar beet is one of the major crops in temperate regions, only a few studies have conducted a bacterial community ana­lysis of its phytosphere. By using PhyloChip, Mendes *et al.* (2011) reported that *Proteobacteria* was‍ ‍the most dominant phylum followed by *Firmicutes*, *Actinobacteria*, and *Bacteroidetes* in the rhizosphere soil of sugar beet seedlings grown in arable soil, while Hudz and Skivka (2021) showed that the rhizosphere soil bacteria of sugar beet were exclusively dominated by *Proteobacteria* and *Actinobacteria*. Shi *et al.* (2014) demonstrated that the endophytic bacteria of the taproot were exclusively dominated by *Proteobacteria*, particularly *Alphaproteobacteria*, throughout the entire growth stages of sugar beet. By using a metagenomic ana­lysis, Tsurumaru *et al.* (2015) showed that the endophytic bacteria of the taproot were dominated by *Proteobacteria*, particularly *Alphaproteobacteria*, and *Actinobacteria*. In the present study, *Proteobacteria*, *Actinobacteria*, and *Bacteroidetes* were identified as the dominant phyla in the lateral roots of sugar beet (Fig. 2A and Table S2). We previously demonstrated the dominance of these phyla in the lateral roots of sugar beet based on clone library ana­lyses (Okazaki *et al.*, 2021). Consistent with previous findings from community ana­lyses of taproot-associated bacteria, the dominance of *Alphaproteobacteria* was noted in the present study because this class potentially includes a number of beneficial bacterial groups (Garrido-Oter *et al.*, 2018; Okazaki *et al.*, 2021). In contrast to previous studies conducted on the taproot of sugar beet, the dominance of *Bacteroidetes* in the lateral roots and rhizosphere soil indicates the high affinity of this phylum bacteria for the exudates of lateral roots because *Bacteroidetes* is generally considered to be a copiotrophic bacterial group using abundant labile C sources (Fierer *et al.*, 2007). However, since the soil types used in experiments may result in marked differences in rhizosphere bacterial diversities, even at the high taxonomic level, such as the phylum level, as shown by Zachow *et al.* (2014), the findings of bacterial community ana­lyses conducted in different experimental sites need to be carefully compared and interpretated. In addition, since the morphology and physiology of the underground tissues of sugar beet markedly changed during plant growth, time series ana­lyses of the bacterial communities of holistic rhizosphere compartments with different soil types are needed to obtain a more detailed understanding of the characteristics of sugar beet rhizosphere bacteria.

### Seasonal shifts in lateral root-associated bacterial community structures

Examinations of seasonal changes of bacterial community structures in lateral roots revealed significant changes in the relative abundances of small numbers of dominant taxa at high taxonomic levels, from phylum to order, during sugar beet growth (Table 3), and the diversity of the bacterial community increased at low taxonomic levels, from family to ASV, as sugar beet grew (Table 2 and 4). The dominant taxa showing high abundances in July and/or August may contribute to the rapid growth of sugar beet (Fig. S1) and may explain the shifts in bacterial community structures along with PC2 for July and August samples in Fig. 1. Five genera in *Rhizobiales* (*Agrobacterium*, *Bradyrhizobium*, *Devosia*, *Mesorhizobium*, and *Rhizobium*) and four genera in *Sphingomonadales* (*Novosphingobium*, *Sphingobium*, *Sphingomonas*, and *Sphingopyxis*) detected as LR bacteria in the present study (Table 4) are all known to include a number of beneficial bacterial groups for plant growth promotion (Antoun *et al.*, 1998; Vurukonda *et al.*, 2016; Zhang *et al.*, 2016; Garrido-Oter *et al.*, 2018; Okazaki *et al.*, 2021; Chhetri *et al.*, 2022). *S. azotifigens* (Table 2 and 4) is also expected to have the potential to promote plant growth as a nitrogen-fixing bacterium in roots (Xie and Yokota, 2006). Although the ecological role of *Caulobacterales* in the roots is largely unknown and plant growth-promoting effects have not been reported for *C. henricii* or *A. biprosthecium*, isolates belonging to the genera *Caulobacter* and *Asticcacaulis* were recently shown to exert these effects (Luo *et al.*, 2019; Yang *et al.*, 2019; Berrios, 2021; Okazaki *et al.*, 2021).

Since four orders (*Burkholderiales*, *Pseudomonadales*, *Actinomycetales*, *Sphingobacteriales*, and *Flavobacteriales*) listed in Table 3 are known to exhibit high antagonistic activity against several plant pathogens as well as plant growth-promoting effects (O’Sullivan and O’Gara, 1992; Doumbou *et al.*, 2001; Francis *et al.*, 2014; Yin *et al.*, 2021), the bacterial members of these orders may play an important role in the protection of sugar beet, particularly in the early growth stage (seedling stage), which is considered to be vulnerable to diverse pathogens and abiotic stresses. Changes in the abundances of major taxa in these orders (showing high abundances in June) may explain the shifts in bacterial community structures along with PC1 in Fig. 1. Two peaks of high relative abundance of *Burkholderiales* in the early growth stage (June and July) and sugar accumulation stage (September) suggest the presence of diverse members with ecologically different functions in this order, such as the high abundances of *Oxalobacteraceae* and *Comamonadaceae* in the early growth stage (June and July) and September, respectively, as shown in Table 4. *Janthinobacterium* belonging to *Oxalobacteraceae* in the wheat rhizosphere was recently associated with disease suppression against *Rhizoctonia solani* (Dilla-Ermita *et al.*, 2021), and a *Janthinobacterium* bacterium derived from the wheat rhizosphere was found to exhibit antagonistic activity against *Pythium* and *Rhizoctonia* spp. (Yin *et al.*, 2021). In the present study, one dominant ASV (ASV_022 in Table 2) corresponding to unclassified *Oxalobacteraceae* was iden­tified and the representative sequence showed 100% iden­tity‍ ‍to species of *Herbaspirillum*, *Herminiimonas*, and *Oxalicibacterium* (Table S5). These three genera were shown to have intimate relationships with sugar beet; however, direct evidence for plant growth-promoting effects has only been obtained for *Herbaspirillum* (Monteiro *et al.*, 2012). Bacteria belonging to *Herbaspirillum* and *Herminiimonas* were isolated from the taproot of sugar beet (Okazaki *et al.*, 2014). *Oxalicibacterium* has been reported as a leaf endophyte associated with the yield increase of sugar beet (Della Lucia *et al.*, 2021). Two dominant ASVs (ASV_040 and ASV_025 in Table 2) belonging to *Comamonadaceae* were identified in the present study. Blast ana­lyses revealed that the representative sequence of ASV_040 showed 100% identity to the sequences of *Rhizobacter* and *Methylibium* (Table S5). While the type species of *Rhizobacter* is known as a plant pathogen (Goto, 2015), the ecological significance of the interaction between plants and other species in this genus remains unclear. Regarding *Methylibium*, an isolate belonging to this genus was shown to exert plant growth-promoting effects (Santiago *et al.*, 2017). A representative sequence of ASV_025 showed 100% identity to *Polaromonas* and *Variovorax*. Isolates belonging to these genera have been reported to exert plant growth-promoting effects on sugar beet (Okazaki *et al.*, 2021).

In the present study, *Streptomyces* and *Pseudomonas* were also highly abundant in June and July (Table 2 and 4), both of which are considered to be more vulnerable plant growth stages to pathogens, such as *Rhizoctonia*, than the later months (August and September). Therefore, the presence of these taxa in the rhizosphere may be important for protecting the young vulnerable seedlings of sugar beet from damping-off caused by fungal pathogens. *Pseudomonas* and *Streptomyces* species have been reported to exhibit strong antagonistic activity against *R. solani* (Zachow *et al.*, 2008; Postma *et al.*, 2010). Mendes *et al.* (2011) also showed that the abundances of *Burkholderiaceae*, *Pseudomonadaceae*, and *Actinobacteria* were strongly associated with the suppression of root rot caused by *R. solani* in the rhizosphere of sugar beet seedlings. One dominant ASV identified for *Streptomyces* (ASV_001) in the present study shared 100% identity with *Streptomyces scabiei* and *Streptomyces turgidiscabies* (Table S5). Consistent with these results, sugar beet has been reported as an alternate host for *S. turgidiscabie*, and, thus, potato production following sugar beet cultivation may cause an outbreak of potato common scab, as reported by Sakuma *et al.* (2011). Besides *Streptomyces*, three genera in *Actinomycetales* (*Kribbella*, *Kutzneria*, and *Amycolatopsis*) were identified as dominant genera (Table 2 and 4). Some endophytic isolates belonging to *Kribbella* have been reported to possess plant growth-promoting traits and exhibit antagonistic activity against several fungal pathogens (Borah and Thakur, 2020). Zhang *et al.* (2022) recently reported the disease-suppressive ability of *Kribbella* through its activation of the plant immune system (Zhang *et al.*, 2022). Plant growth-promoting effects and antagonistic activity against fungal pathogens have also been demonstrated for *Kutzneria* (Devi *et al.*, 2021) and *Amycolatopsis* (Alekhya and Gopalakrishnan, 2016; Gopalakrishnan *et al.*, 2019; Cabrera *et al.*, 2020). *Pedobacter* and *Flavobacterium* were identified in the present study as the dominant genera in *Sphingobacteriales* and *Flavobacteriales*, respectively. These genera have also been shown to exert plant growth-promoting effects and exhibit antagonistic activity against fungal pathogens (de Boer *et al.*, 2007; Yin *et al.*, 2013; Kolton *et al.*, 2016; Morais *et al.*, 2019). The high abundances of potential antagonistic taxa at the initial growth stage of sugar beet, as described above, are considered to be very important for protecting vulnerable young seedlings from pathogens, and this appears to be consistent with the speculation that the rhizosphere of a modern cultivar of sugar beet has the ability to enrich a higher antagonistic potential than that of wild beet, as reported by Zachow *et al.* (2014).

In contrast to the orders described above, increases in the relative abundances of *Chloroflexi*, *Cytophagales*, *Ellin329*, *Myxococcales*, *Saprospirales*, *Verrucomicrobia*, and *Xanthomonadales* in August and/or September appeared to be associated with the accumulation of organic matter in the mature or senescence rhizosphere. Therefore, the majority of known bacteria in these orders are considered not only to aggressively interact with plants, but also passively associate with plants through the degradation of plant-derived organic matter in the rhizosphere. Bacteria in the rhizosphere of young plants preferentially utilize simple low-mole­cular-weight compounds, such as amino acids, whereas bacteria in the rhizosphere of mature plants efficiently utilize more recalcitrant organic matter, including high-mole­cular-weight carbohydrates (Houlden *et al.*, 2008). Bacterial members of *Chloroflexi*, *Ellin329*, and *Verrucomicrobia* are well-known degraders of organic matter derived from plant tissues under anaerobic conditions (Chin *et al.*, 2001; Podosokorskaya *et al.*, 2013; Harbison *et al.*, 2016). The high abundance of *Myxococcales*, including *Haliangiaceae*, in September most likely reflects the high activity of their bacterial predation in the mature rhizosphere, which harbors a high microbial biomass. As previously reported by Wang *et al.* (2020) for bulk soils of arable lands, the relative abundance of *Myxococcales* in the present study also positively correlated with diversity indexes (the number of ASVs, Chao1, ACE, and the Shannon index in Fig. S3 and S4), indicating that *Myxococcales* is also important for regulating bacterial community structures in the rhizosphere of sugar beet. Besides bacterial predation, some members of *Myxococcales* have the ability to decompose diverse organic matter (Dworkin, 1966; Reichenbach, 2015), and this trait may be advantageous for proliferation in the organic-rich, mature rhizosphere. Several bacterial members in *Cytophagales* and *Saprospirales* are well-known degraders of plant-derived organic macromolecules under aerobic conditions (Hargreaves *et al.*, 2015; Nakagawa, 2015). In *Cytophagles*, the high abundance of *Cytophagaceae* in September (Table 4) may be related to the increase in the microbial biomass in the mature rhizosphere because this family are bacterial predators (Pérez *et al.*, 2016). In *Saprospirales*, *Chitinophagaceae* appeared to be one of the highly dominant taxa throughout all of the growth stages investigated (Table 4). *C. arvensicola* exhibits the ability to degrade plant-derived polysaccharides, such as xylan and laminarin (Pankratov *et al.*, 2006). Although the potential ability for biological control based on chitinase activity has been discussed for *Chitinophaga*, this plant growth-promoting effects of this genus have not yet been confirmed. The high diversities of *Cytophagaceae* and unclassified *Chitinophagaceae* at the ASV levels and the complex patterns for the dynamics of their relative abundances observed in the present study (Table S4) imply the high ecological adaptability of these taxa for utilizing diverse organic compounds in the rhizosphere, which may be related to the formation of the loose cluster in September samples in Fig. 1.

While the order of *Xanthomonadales* includes a number of major plant pathogens, it also harbors diverse bacterial groups with various ecological characteristics most likely due to the early divergence of this bacterial group within the class *Gammaproteobacteria* (Naushad *et al.*, 2015). Therefore, it is difficult to speculate or discuss a representative ecological feature for *Xanthomonadales* based on simple relative abundance data at the order level. The relative abundance of *Rhodanobacter* was the highest in June and the lowest in September, while the relative abundances of *Steroidobacter* and *Dokdonella* showed the opposite trend. *Rhodanobacter* has been reported to exert plant growth-promoting effects (Thijs *et al.*, 2014) and has also been shown to exhibit antagonistic activity against fungal pathogens (de Clercq *et al.*, 2006; Shin *et al.*, 2007; Huo *et al.*, 2018). The gradual increase observed in the relative abundance of *Steroidobacter* between June and September may have contributed to a reduction in several environmental stresses and promoted the growth of sugar beet because the relative abundance of this genus in the rhizosphere was previously shown to positively correlate with the disease suppressiveness of soils (Vida *et al.*, 2016; Wang *et al.*, 2019; Mayerhofer *et al.*, 2021). Furthermore, *Steroidobacter* has been shown to induce stem and root elongation by alleviating environmental stresses with the production of brassinosteroids in the rhizosphere (Zarraonaindia *et al.*, 2015). Although *Dokdonella* has been identified as an active member of the rhizosphere (Gkarmiri *et al.*, 2017), the ecological role of this genera in the phytosphere remains unknown. The shifts in bacterial community structures described above suggest that the major taxa showing high abundances in June, July, and August are mainly plant growth promoters, while those in September most likely play an ecological role as degraders of organic matter derived from root senescence.

### Seasonal shifts in beneficial bacterial genera in lateral roots for plant growth promotion

PGPB for sugar beet have been identified in 21 genera (Table S6). The majority of the genera for PGPB isolated from the lateral roots in our previous study (*Asticcacaulis*, *Mesorhizobium*, *Polaromonas*, *Sphingobium*, *Sphingomonas*, and *Sphingopyxis*) (Okazaki *et al.*, 2021) were highly abundant in the active growth period of sugar beet (between June and August) in the present study, suggesting the contribution of these PGPB to the growth of sugar beet under field conditions. Other PGPB in sugar beet, such as *Pseudomonas*, *Flavobacterium*, *Paenibacillus*, and *Bacillus*, also showed high abundances in the early growth stages (June and/or July). The relative abundances of *Burkholderia* and *Lysobacter* were moderate and remained relatively constant between June and September, while the other genera reported for sugar beet PGPB were detected as minor taxa in the present study. These results indicate the high efficiency of the community ana­lysis-based screening of potential bene­ficial microbes for agricultural practice in our previous study (Okazaki *et al.*, 2021).

In conclusion, the time series analyses of bacterial diversity in the lateral roots of sugar beet grown in an Andosol field in Japan revealed that marked seasonal shifts in bacterial community structures occurred in the lateral roots from the late seedling to sugar accumulation stages. Bacterial diversity in the lateral roots was exclusively dominated by only three phyla (*Proteobacteria*, *Actinobacteria*, and *Bacteroidetes*) from the late seedling to sugar accumulation stages of sugar beet. At the lower taxonomic levels, dominant taxa were roughly classified into three groups showing high abundances in the late seedling (June), rapid growth (July and August), and sugar accumulation (September) stages. Among these dominant taxa, the plant growth-promoting effects of 12 genera (*Amycolatopsis*, *Bradyrhizobium*, *Caulobacter*, *Devosia*, *Flavobacterium*, *Janthinobacterium*, *Kribbella*, *Kutzneria*, *Pedobacter*, *Rhizobium*, *Rhodanobacter*, and *Steroidobacter*) warrant further investigation as good candidates for PGPB of sugar beet based on culture-dependent methods because they have beneficial traits as biopesticides and/or biofertilizers for several other crops. The present results clearly indicate that time series ana­lyses of microbial communities during plant growth stages provide valuable information for understanding the ecological roles of plant-associated microbes and accelerating the practical use of the beneficial functions of PGPB under field conditions.

## Citation

Ikeda, S., Okazaki, K., Takahashi, H., Tsurumaru, H., and Minamisawa, K. (2023) Seasonal Shifts in Bacterial Community Structures in the Lateral Root of Sugar Beet Grown in an Andosol Field in Japan. *Microbes Environ ***38**: ME22071.

https://doi.org/10.1264/jsme2.ME22071

## Supplementary Material

Supplementary Material

## Figures and Tables

**Table 1.** Diversity indexes for lateral root-associated bacteria of sugar beet grown in an Andosol experimental field in Japan between June and September

Diversity index^a^	Sampling month^b^								Sig.^c^
	June		July		August		September		
No. of singletons	14.0±12.9	b	39.5±32.6	ab	91.3±33.6	a	72.0±49.1	a	**
No. of ASVs	230.0±64.0	b	332.5±96.3	ab	444.3±78.4	a	440.2±111.2	a	**
Chao1	235.5±69.7	b	353.8±115.4	ab	504.3±107.2	a	484.2±146.8	a	**
ACE	235.5±69.3	b	354.6±119.5	ab	508.3±107.5	a	485.9±150.0	a	**
Shannon	6.4±0.4	b	7.1±0.3	a	7.3±0.2	a	7.5±0.3	a	***
Simpson	0.968±0.016	b	0.984±0.004	a	0.986±0.002	a	0.989±0.002	a	***

^a^ Diversity indexes are calculated based on 5,491 reads per sample. ^b^ Results are shown as the average±S.D. (*n*=6). The same letter indicates no significant difference among sampling months. ^c^ ** and *** indicate a significant difference among sampling months by a one-way ANOVA at *P*<0.01 and *P*<0.001, respectively.

**Table 2.** Sugar beet lateral root-associated bacteria showing significant differences in relative abundances among sampling months at the ASV level

ASV ID^a^	Closest taxon^b^	Sampling month^c^								Sig.^d^
		June		July		August		September		
ASV_001	*Streptomyces* (g)	13.9±5.1	a	7.2±2.7	b	6.0±1.1	b	2.6±1.0	c	***
ASV_003	*Caulobacter henricii*	3.4±0.6	a	3.5±1.1	a	2.3±0.7	a	1.0±0.4	b	***
ASV_030	*Janthinobacterium* (g)	3.0±1.4	a	1.3±0.5	b	0.2±0.1	c	0.2±0.1	c	***
ASV_014	*Pedobacter* (g)	2.8±1.1	a	1.7±0.9	ab	0.8±0.5	bc	0.2±0.4	c	***
ASV_015	*Sphingobium* (g)	2.0±0.9	a	1.1±0.4	a	1.2±0.6	a	0.4±0.2	b	***
ASV_005	*Chitinophaga arvensicola*	1.9±0.7	ab	1.2±0.7	ab	2.4±1.2	a	1.0±0.5	b	*
ASV_019	*Rhodanobacter* (g)	1.9±1.0	a	1.2±0.3	a	0.9±0.3	ab	0.3±0.3	b	***
ASV_022	*Oxalobacteraceae* (f)	1.8±0.7	a	1.5±0.5	a	0.7±0.2	b	0.3±0.2	b	***
**ASV_008**	***Niastella*** ** (g)**	1.8±0.9		1.5±0.5		0.9±0.4		2.0±1.0		—
ASV_012	*Kribbella* (g)	1.8±0.7	a	1.5±0.4	a	1.2±0.5	ab	0.7±0.4	b	**
ASV_006	*Rhizobium* (g)	1.8±0.6	ab	1.5±0.4	ab	2.2±0.4	a	1.2±0.3	b	**
ASV_020	*Pseudomonas* (g)	1.4±1.2	ab	2.1±2.0	a	0.2±0.1	c	0.4±0.3	bc	**
ASV_038	*Kribbella* (g)	1.4±0.3	a	1.0±0.5	ab	0.5±0.2	bc	0.2±0.3	c	***
ASV_002	*Chitinophagaceae* (f)	1.2±0.4	b	3.4±1.1	a	3.4±2.0	a	2.0±1.0	ab	**
ASV_039	*Streptomyces* (g)	1.2±0.3	a	0.7±0.2	b	0.6±0.3	b	0.4±0.2	b	***
ASV_007	*Sphingomonas* (g)	1.2±0.5	b	1.1±0.3	b	2.5±0.8	a	0.9±0.9	b	**
ASV_064	*Streptomycetaceae* (f)	1.2±0.4	a	0.5±0.3	ab	0.2±0.2	b	0.1±0.2	b	***
ASV_032	*Asticcacaulis biprosthecium*	1.1±0.2	a	1.1±0.4	a	0.8±0.4	a	0.1±0.2	b	***
**ASV_033**	***Niastella*** ** (g)**	1.0±0.3		0.8±0.5		0.8±0.2		0.5±0.1		—
ASV_004	*Bradyrhizobium* (g)	0.9±0.3	b	1.8±0.5	a	2.3±0.4	a	2.2±0.7	a	***
ASV_023	*Novosphingobium* (g)	0.8±0.6	ab	0.5±0.3	ab	1.4±0.9	a	0.2±0.3	b	**
**ASV_018**	***Devosia*** ** (g)**	0.8±0.2		1.1±0.4		1.0±0.2		0.9±0.4		—
ASV_016	*Sphingomonas azotifigens*	0.7±0.2	b	1.6±0.4	a	0.9±0.4	b	0.9±0.5	ab	**
ASV_009	*Agrobacterium* (g)	0.4±0.1	b	1.2±0.4	a	1.8±1.0	a	1.1±0.4	a	***
**ASV_031**	***Sphingobacteriaceae*** ** (f)**	0.4±0.2		1.0±1.2		0.8±0.5		0.6±0.2		—
ASV_026	*Steroidobacter* (g)	0.3±0.1	b	0.5±0.2	b	0.6±0.4	b	1.8±0.3	a	***
ASV_024	*Sinobacteraceae* (f)	0.2±0.2	b	0.5±0.2	b	0.4±0.1	b	1.9±0.8	a	***
ASV_010	*Novosphingobium* (g)	0.2±0.3	b	0.8±0.6	ab	1.3±0.5	a	1.8±0.8	a	***
ASV_034	*Kutzneria* (g)	0.2±0.2	b	0.3±0.2	b	1.2±0.3	a	0.8±0.8	ab	**
ASV_021	*Chitinophaga* (g)	0.2±0.3	b	0.3±0.2	ab	0.7±0.3	ab	2.0±3.0	a	*
ASV_054	*Gammaproteobacteria* (c)	0.2±0.4	b	0.1±0.2	b	0.0±0.1	b	1.9±1.7	a	***
ASV_017	*Amycolatopsis* (g)	0.2±0.1	b	0.4±0.2	b	1.1±0.2	a	1.3±0.5	a	***
ASV_027	*Dokdonella* (g)	0.1±0.1	c	0.5±0.1	b	1.0±0.2	a	0.9±0.4	ab	***
ASV_040	*Comamonadaceae* (f)	0.1±0.2	c	0.2±0.2	bc	0.4±0.2	b	1.3±0.6	a	***
ASV_025	*Comamonadaceae* (f)	0.1±0.2	c	0.5±0.2	b	0.5±0.3	b	2.1±0.5	a	***
ASV_013	*Novosphingobium* (g)	0.1±0.2	c	0.5±0.4	bc	1.8±1.3	a	0.6±0.5	b	***
ASV_011	*Steroidobacter* (g)	0.0±0.0	c	0.1±0.1	c	0.7±0.3	b	3.2±1.2	a	***
ASV_046	*Gammaproteobacteria* (c)	0.0±0.0	b	0.0±0.1	b	0.1±0.1	b	1.1±1.0	a	***

^a^ ASVs with relative abundances of 1% or more than 1% in any one of the sampling months are shown. Relative abundance was calculated based on 5,491 reads per sample. ASVs indicated in bold font showed no significant differences in relative abundance among the sampling months. ^b^ Letters (c, f, and g) in parentheses indicate taxonomic units (class, family, and genus, respectively). ^c^ Results are shown as the average±S.D. (*n*=6). The same letter indicates no significant difference among the months, and months with high, low, and moderate abundances are highlighted with dark gray, white, and light gray backgrounds, respectively. ^d^ *, **, and *** indicate a significant difference among sampling months by a one-way ANOVA at *P*<0.05, *P*<0.01, and *P*<0.001, respectively. “—” indicates no significant difference among the sampling months.

**Table 3.** Sugar beet lateral root-associated bacteria showing significant differences in relative abundances (%) among sampling months at phylum, class, and order levels

Taxon^a^	Sampling month^b^								Sig.^c^
	June		July		August		September		
*Proteobacteria*	50.3±5.4	b	54.3±2.1	ab	56.1±3.6	ab	58.7±5.5	a	*
*Alphaproteobacteria*	26.1±2.0	bc	30.5±2.5	b	37.0±5.7	a	23.7±3.0	c	***
*Rhizobiales*	11.6±1.2	b	13.8±1.3	a	15.2±1.4	a	10.6±1.6	b	***
*Sphingomonadales*	8.5±1.2	b	9.5±0.9	b	15.8±3.9	a	8.5±1.4	b	***
*Caulobacterales*	5.5±0.8	a	6.0±1.6	a	4.2±0.9	a	2.1±0.6	b	***
*Ellin329*	0.3±0.1	b	0.7±0.3	ab	1.2±0.7	a	1.1±0.3	a	***
*Betaproteobacteria*	12.6±3.1	a	11.7±1.7	a	7.5±1.4	b	11.3±1.8	a	**
*Burkholderiales*	11.7±3.1	a	11.0±1.7	a	6.9±1.3	b	10.0±1.4	a	**
*Gammaproteobacteria*	10.8±3.3	b	11.3±2.1	b	9.8±1.0	b	18.8±3.6	a	***
*Xanthomonadales*	5.5±1.2	c	7.0±1.1	bc	8.7±1.3	b	13.1±2.4	a	***
*Pseudomonadales*	4.1±2.3	a	3.4±2.1	a	0.6±0.4	b	0.8±0.5	b	***
Unclassified *Gammaproteobacteria* (c)	0.5±0.7	b	0.4±0.3	b	0.2±0.1	b	3.6±2.2	a	***
*Deltaproteobacteria*	0.9±0.5	b	0.8±0.3	b	1.7±0.7	b	4.8±2.0	a	***
*Myxococcales*	0.8±0.4	b	0.6±0.2	b	1.5±0.6	b	4.5±1.9	a	***
*Actinobacteria*	25.3±6.9	a	16.7±4.3	b	16.1±2.0	bc	10.5±3.3	c	***
*Actinomycetales*	25.0±6.9	a	16.4±4.4	b	15.6±2.1	b	10.2±3.3	b	***
*Verrucomicrobia*	0.5±0.3	b	0.8±0.4	b	0.9±0.3	b	2.0±0.7	a	***
*Chloroflexi*	0.4±0.2	b	0.3±0.2	b	0.6±0.2	b	1.2±0.6	a	***
*Bacteroidetes*	20.5±2.9		25.1±3.0		22.6±2.6		23.0±5.1		—
*Saprospirales*	12.0±1.4	b	16.8±1.7	a	18.0±3.2	a	17.8±4.7	a	**
*Sphingobacteriales*	6.2±1.2	a	5.9±2.2	a	3.6±0.7	b	2.5±0.5	b	***
*Flavobacteriales*	1.3±0.6	a	1.4±0.6	a	0.4±0.1	b	0.9±0.5	ab	**
*Cytophagales*	1.0±0.3	b	1.0±0.2	b	0.7±0.2	b	1.7±0.6	a	***

^a^ Taxa with relative abundances of 1% or more than 1% in any one of the sampling months are shown. When the same value for relative abundance is obtained at different taxonomic levels for a bacterial group, only the lowest taxonomic group is shown. Relative abundance was calculated based on 5,491 reads per sample. Letters (c) in parentheses indicate taxonomic units (class). *Bacteroidetes* showed no significant differences in relative abundance among the sampling months, but is listed as a reference for the order of this phylum. ^b^ Results are shown as the average±S.D. (*n*=6). The same letter indicates no significant difference among the months, and months with high, low, and moderate abundances are highlighted with dark gray, white, and light gray backgrounds, respectively. ^c^ *, **, and *** indicate a significant difference among months by a one-way ANOVA at *P*<0.05, *P*<0.01, and *P*<0.001, respectively. “—” indicates no significant difference among the sampling months.

**Table 4.** Sugar beet lateral root-associated bacteria showing significant differences in relative abundances (%) among sampling months at family, genus, and species levels

Taxon^a^	Sampling month^b^								Sig.^c^
	June		July		August		September		
*Streptomycetaceae*	17.7±6.4	a	9.1±3.0	b	7.6±1.5	b	3.6±1.3	c	***
*Streptomyces*	15.7±5.7	a	8.3±3.0	b	7.0±1.4	b	3.4±1.2	c	***
Unclassified *Streptomycetaceae* (f)	2.0±1.1	a	0.8±0.5	b	0.6±0.2	b	0.2±0.3	b	***
*Chitinophagaceae* ^d^	11.9±1.4	b	16.7±1.7	a	17.9±3.2	a	17.7±4.7	a	**
Unclassified *Chitinophagaceae* (f)’	4.7±0.6	b	7.8±1.1	a	7.6±2.0	a	5.9±1.0	ab	***
Unclassified *Chitinophagaceae* (f)”	0.7±0.2	c	1.6±0.5	b	1.8±0.5	ab	2.5±0.6	a	***
*Chitinophaga arvensicola*	1.9±0.7	ab	1.2±0.7	ab	2.4±1.2	a	1.0±0.5	b	*
*Oxalobacteraceae*	8.5±1.7	a	5.1±0.8	b	1.7±0.3	c	0.9±0.1	d	***
*Janthinobacterium*	3.9±1.3	a	1.5±0.7	b	0.3±0.1	c	0.2±0.1	c	***
Unclassified *Oxalobacteraceae* (f)	2.8±0.7	a	2.5±0.4	a	0.9±0.2	b	0.6±0.1	b	***
*Sphingomonadaceae*	8.3±1.1	b	9.0±0.7	b	14.8±3.5	a	8.2±1.3	b	***
*Sphingobium*	2.3±1.0	a	1.3±0.4	b	1.2±0.6	b	0.5±0.2	c	***
*Novosphingobium*	2.0±0.6	c	2.5±0.3	c	7.1±2.9	a	4.4±0.7	b	***
*Sphingomonas*	1.9±0.6	c	3.8±0.8	ab	4.4±0.8	a	2.7±1.3	bc	***
*Sphingomonas azotifigens*	0.7±0.2	b	1.6±0.4	a	0.9±0.4	b	0.9±0.5	ab	**
*Sphingopyxis*	1.4±0.8	a	0.9±0.5	a	0.9±0.4	a	0.1±0.1	b	***
*Sphingobacteriaceae*	6.2±1.2	a	5.7±2.2	a	3.5±0.7	b	1.9±0.7	b	***
*Pedobacter*	4.6±1.3	a	3.2±1.3	ab	1.8±0.8	b	0.6±0.4	c	***
*Caulobacteraceae*	5.5±0.8	a	6.0±1.6	a	4.2±0.9	a	2.1±0.6	b	***
*Caulobacter*	3.6±0.6	ab	3.9±1.2	a	2.5±0.8	b	1.3±0.4	c	***
*Caulobacter henricii*	3.6±0.6	a	3.6±1.1	ab	2.3±0.7	b	1.0±0.4	c	***
*Asticcacaulis biprosthecium*	1.1±0.2	a	1.1±0.4	a	0.8±0.4	a	0.1±0.2	b	***
*Rhizobiaceae*	4.6±0.8	ab	5.0±0.8	a	5.9±1.1	a	3.6±0.5	b	***
*Rhizobium*	3.0±1.1	a	2.7±0.7	a	3.5±0.5	a	1.6±0.4	b	**
*Agrobacterium*	0.5±0.2	b	1.2±0.4	a	1.8±1.0	a	1.2±0.4	ab	**
*Pseudomonas*	4.1±2.3	a	3.4±2.1	a	0.6±0.4	b	0.8±0.6	b	***
*Nocardioidaceae*	4.1±0.9	a	3.4±0.7	ab	2.3±0.8	bc	1.3±0.8	c	***
*Kribbella*	3.6±0.8	a	3.0±0.7	ab	2.0±0.8	bc	1.2±0.8	c	***
*Rhodanobacter*	2.5±1.2	a	2.2±0.7	ab	1.9±1.1	ab	0.9±0.8	b	*
*Bradyrhizobiaceae*	2.3±0.3	b	3.5±0.5	a	4.1±0.7	a	3.1±0.8	ab	***
Unclassified *Bradyrhizobiaceae* (f)	1.3±0.7	a	1.3±0.2	a	1.3±0.2	a	0.4±0.2	b	***
*Bradyrhizobium*	0.9±0.3	b	2.0±0.4	a	2.7±0.5	a	2.5±0.8	a	***
*Devosia*	2.2±0.5	ab	2.4±0.7	a	1.7±0.6	ab	1.5±0.2	b	*
*Comamonadaceae*	2.1±1.1	c	4.8±1.0	b	4.0±1.0	b	8.2±1.9	a	***
Unclassified *Comamonadaceae* (f)	1.7±0.8	c	3.5±0.8	b	3.0±1.0	b	6.2±1.5	a	***
*Phyllobacteriaceae*	1.7±0.4	a	2.1±0.3	a	1.9±0.4	a	0.9±0.3	b	***
*Mesorhizobium*	1.3±0.2	a	1.6±0.2	a	1.5±0.4	a	0.7±0.2	b	***
*Cytophagaceae*	1.0±0.3	b	1.0±0.2	b	0.7±0.2	b	1.7±0.6	a	***
*Flavobacterium*	0.9±0.5	a	1.1±0.6	a	0.2±0.1	b	0.4±0.5	ab	**
*Actinosynnemataceae*	0.8±0.3	b	1.1±0.4	b	2.2±0.2	a	1.9±0.9	a	***
*Kutzneria*	0.2±0.2	b	0.3±0.2	b	1.2±0.3	a	0.8±0.8	ab	**
*Sinobacteraceae*	0.6±0.2	c	1.5±0.5	b	2.4±0.6	b	8.9±2.0	a	***
*Steroidobacter*	0.3±0.1	d	0.7±0.2	c	1.6±0.5	b	5.9±1.0	a	***
Unclassified *Sinobacteraceae* (f)	0.3±0.1	c	0.7±0.3	b	0.8±0.1	b	3.0±1.1	a	***
*Dokdonella*	0.3±0.1	c	0.9±0.1	b	1.6±0.3	a	1.5±0.5	a	***
Unclassified *Gammaproteobacteria* (c)	0.5±0.7	b	0.4±0.3	b	0.2±0.1	b	3.6±2.2	a	***
Unclassified *Ellin329* (o)	0.3±0.1	b	0.7±0.3	ab	1.2±0.7	a	1.1±0.3	a	***
*Haliangiaceae*	0.2±0.1	b	0.2±0.2	b	0.4±0.2	b	1.4±0.9	a	***
*Amycolatopsis*	0.2±0.1	b	0.4±0.2	b	1.1±0.2	a	1.3±0.5	a	***
Unclassified *Myxococcales* (o)	0.1±0.1	c	0.1±0.1	c	0.4±0.2	b	2.2±0.7	a	***

^a^ Taxa with relative abundances of 1% or more than 1% in any one of the sampling months are shown. Relative abundance was calculated based on 5,491 reads per sample. Letters (f, c, and o) in parentheses indicate taxonomic units (family, class, and order, respectively). ^b^ Results are shown as the average±S.D. (*n*=6). The same letter indicates no significant difference among the months, and months with high, low, and moderate abundances are highlighted with dark gray, white, and light gray backgrounds, respectively. ^c^ *, **, and *** indicate a significant difference among months by a one-way ANOVA at *P*<0.05, *P*<0.01, and *P*<0.001, respectively. ^d^ Unclassified Chitinophagaceae (f)’ and (f)” stand for “f__Chitinophagaceae;g__” and “f__Chitinophagaceae;__”, respectively, as outputs with taxonomic ana­lyses at the genus level.

## References

[B1] Alekhya, G., and Gopalakrishnan, S. (2016) Exploiting plant growth-promoting *Amycolatopsis* sp. in chickpea and sorghum for improving growth and yield. J Food Legumes 29: 225–231.

[B2] Antoun, H., Beauchamp, C.J., Goussard, N., Chabot, R., and Lalande, R. (1998) Potential of *Rhizobium* and *Bradyrhizobium* species as plant growth promoting rhizobacteria on non-legumes: effect on radishes (*Raphanus sativus* L.). In *Molecular Microbial Ecology of the Soil*. Hardarson, G., and Broughton, W.J. (eds). Dordrecht: Springer, pp. 57–67.

[B3] Berrios, L. (2021) Complete genome sequence of the plant-growth-promoting bacterium *Caulobacter segnis* CBR1. Curr Microbiol 78: 2935–2942. 34047832 10.1007/s00284-021-02548-z

[B4] Bolyen, E., Rideout, J.R., Dillon, M.R., Bokulich, N.A., Abnet, C.C., Al-Ghalith, G.A., et al. (2019) Reproducible, interactive, scalable and extensible microbiome data science using QIIME 2. Nat Biotechnol 37: 852–857. 31341288 10.1038/s41587-019-0209-9 PMC7015180

[B90] Borah, A., and Thakur, D. (2020) Phylogenetic and functional characterization of culturable endophytic actinobacteria associated with *Camellia* spp. for growth promotion in commercial tea cultivars. Front Microbiol 11: 318. 32180767 10.3389/fmicb.2020.00318 PMC7059647

[B5] Bulgari, D., Montagna, M., Gobbi, E., and Faoro, F. (2019) Green technology: bacteria-based approach could lead to unsuspected microbe–plant–animal interactions. Microorganisms 7: 44. 30736387 10.3390/microorganisms7020044 PMC6406919

[B6] Cabrera, R., García-López, H., Aguirre-von-Wobeser, E., Orozco-Avitia, J.A., and Gutiérrez-Saldaña, A.H. (2020) *Amycolatopsis* BX17: An actinobacterial strain isolated from soil of a traditional milpa agroecosystem with potential biocontrol against *Fusarium graminearum*. Biol Control 147: 104285.

[B7] Çakmakçi, R., Kantar, F., and Algur, Ö.F. (1999) Sugar beet and barley yields in relation to *Bacillus polymyxa* and *Bacillus megaterium* var. *phosphaticum* inoculation. J Plant Nutr Soil Sci 162: 437–442.

[B8] Çakmakçi, R., Kantar, F., and Sahin, F. (2001) Effect of N_2_-fixing bacterial inoculations on yield of sugar beet and barley. J Plant Nutr Soil Sci 164: 527–531.

[B9] Çakmakçi, R., Dönmez, F., Aydın, A., and Şahin, F. (2006) Growth promotion of plants by plant growth-promoting rhizobacteria under greenhouse and two different field soil conditions. Soil Biol Biochem 38: 1482–1487.

[B10] Caporaso, J.G., Kuczynski, J., Stombaugh, J., Bittinger, K., Bushman, F.D., Costello, E.K., et al. (2010) QIIME allows ana­lysis of high-throughput community sequencing data. Nat Methods 7: 335–336. 20383131 10.1038/nmeth.f.303 PMC3156573

[B11] Caporaso, J.G., Lauber, C.L., Walters, W.A., Berg-Lyons, D., Lozupone, C.A., Turnbaugh, P.J., et al. (2011) Global patterns of 16S rRNA diversity at a depth of millions of sequences per sample. Proc Natl Acad Sci U S A 108: 4516–4522. 20534432 10.1073/pnas.1000080107 PMC3063599

[B12] Chhetri, G., Kim, I., Kang, M., Kim, J., So, Y., and Seo, T. (2022) *Devosia rhizoryzae* sp. nov., and *Devosia* *oryziradicis* sp. nov., novel plant growth promoting members of the genus *Devosia*, isolated from the rhizosphere of rice plants. J Microbiol 60: 1–10. 34826099 10.1007/s12275-022-1474-8

[B13] Chin, K.J., Liesack, W., and Janssen, P.H. (2001) *Opitutus terrae* gen. nov., sp. nov., to accommodate novel strains of the division ‘*Verrucomicrobia*’ isolated from rice paddy soil. Int J Syst Evol Microbiol 51: 1965–1968. 11760935 10.1099/00207713-51-6-1965

[B14] Compant, S., Clément, C., and Sessitsch, A. (2010) Plant growth-promoting bacteria in the rhizo-and endosphere of plants: their role, colonization, mechanisms involved and prospects for utilization. Soil Biol Biochem 42: 669–678.

[B15] de Boer, W., Wagenaar, A.M., Klein Gunnewiek, P.J., and van Veen, J.A. (2007) *In vitro* suppression of fungi caused by combinations of apparently non-antagonistic soil bacteria. FEMS Microbiol Ecol 59: 177–185. 17233750 10.1111/j.1574-6941.2006.00197.x

[B16] de Clercq, D., Van Trappen, S., Cleenwerck, I., Ceustermans, A., Swings, J., Coosemans, J., and Ryckeboer, J. (2006) *Rhodanobacter spathiphylli* sp. nov., a gammaproteobacterium isolated from the roots of *Spathiphyllum* plants grown in a compost-amended potting mix. Int J Syst Evol Microbiol 56: 1755–1759. 16902003 10.1099/ijs.0.64131-0

[B17] de Vries, S.C., van de Ven, G.W., van Ittersum, M.K., and Giller, K.E. (2010) Resource use efficiency and environmental performance of nine major biofuel crops, processed by first-generation conversion techniques. Biomass Bioenergy 34: 588–601.

[B18] Della Lucia, M.C., Bertoldo, G., Broccanello, C., Maretto, L., Ravi, S., Marinello, F., et al. (2021) Novel effects of leonardite-based applications on sugar beet. Front Plant Sci 12: 646025. 33815453 10.3389/fpls.2021.646025 PMC8013720

[B19] Devi, T.S., Vijay, K., Vidhyavathi, R.M., Kumar, P., Govarthanan, M., and Kavitha, T. (2021) Antifungal activity and mole­cular docking of phenol, 2,4-bis (1,1-dimethylethyl) produced by plant growth-promoting actinobacterium *Kutzneria* sp. strain TSII from mangrove sediments. Arch Microbiol 203: 4051–4064. 34046705 10.1007/s00203-021-02397-1

[B20] Dilla-Ermita, C.J., Lewis, R.W., Sullivan, T.S., and Hulbert, S.H. (2021) Wheat genotype-specific recruitment of rhizosphere bacterial microbiota under controlled environments. Front Plant Sci 12: 718264. 34925393 10.3389/fpls.2021.718264 PMC8671755

[B21] Doumbou, C.L., Hamby Salove, M.K., Crawford, D.L., and Beaulieu, C. (2001) Actinomycetes, promising tools to control plant diseases and to promote plant growth. Phytoprotection 82: 85–102.

[B22] Dunne, C., Moënne-Loccoz, Y., McCarthy, J., Higgins, P., Powell, J., Dowling, D.N., and O’gara, F. (1998) Combining proteolytic and phloroglucinol-producing bacteria for improved biocontrol of *Pythium*-mediated damping-off of sugar beet. Plant Pathol 47: 299–307.

[B23] Dworkin, M. (1966) Biology of the myxobacteria. Annu Rev Microbiol 20: 75–106. 5330241 10.1146/annurev.mi.20.100166.000451

[B24] Fierer, N., Bradford, M.A., and Jackson, R.B. (2007) Toward an ecological classification of soil bacteria. Ecology 88: 1354–1364. 17601128 10.1890/05-1839

[B25] Francis, I.M., Jochimsen, K.N., de Vos, P., and van Bruggen, A.H. (2014) Reclassification of rhizosphere bacteria including strains causing corky root of lettuce and proposal of *Rhizorhapis suberifaciens* gen. nov., comb. nov., *Sphingobium mellinum* sp. nov., *Sphingobium xanthum* sp. nov. and *Rhizorhabdus argentea* gen. nov., sp. nov. Int J Syst Evol Microbiol 64: 1340–1350. 24436067 10.1099/ijs.0.058909-0

[B26] Garrido-Oter, R., Nakano, R.T., Dombrowski, N., Ma, K.W., Team, T.A., McHardy, A.C., et al. (2018) Modular traits of the Rhizobiales root microbiota and their evolutionary relationship with symbiotic rhizobia. Cell Host Microbe 24: 155–167. 30001518 10.1016/j.chom.2018.06.006 PMC6053594

[B27] Gkarmiri, K., Mahmood, S., Ekblad, A., Alström, S., Högberg, N., and Finlay, R. (2017) Identifying the active microbiome associated with roots and rhizosphere soil of oilseed rape. Appl Environ Microbiol 83: e01938-17. 28887416 10.1128/AEM.01938-17 PMC5666129

[B28] Godshall, M.A. (2012) Sugar and other sweeteners. In *Handbook of Industrial Chemistry and Biotechnology*. Kent, J.A. (ed.) New York, NY: Springer Science+Business Media, pp. 1403–1430.

[B29] Gopalakrishnan, S., Srinivas, V., Naresh, N., Alekhya, G., and Sharma, R. (2019) Exploiting plant growth-promoting *Amycolatopsis* sp. for bio-control of charcoal rot of sorghum (*Sorghum bicolor* L.) caused by *Macrophomina phaseolina* (Tassi) Goid. Arch Phytopathol Pflanzenschutz 52: 543–559.

[B30] Goto, M. (2015) *Rhizobacter*. In *Bergey’s Manual of Systematics of Archaea and Bacteria*. Hoboken, NJ: John Wiley & Sons, pp. 1–5.

[B31] Hara, S., Matsuda, M., and Minamisawa, K. (2019) Growth stage-dependent bacterial communities in soybean plant tissues: *Methylorubrum* transiently dominated in the flowering stage of soybean shoot. Microbes Environ 34: 446–450. 31413227 10.1264/jsme2.ME19067 PMC6934392

[B32] Harbison, A.B., Carson, M.A., Lamit, L.J., Basiliko, N., and Bräuer, S.L. (2016) A novel isolate and widespread abundance of the candidate alphaproteobacterial order (Ellin 329), in southern Appalachian peatlands. FEMS Microbiol Lett 363: fnw151. 27302469 10.1093/femsle/fnw151

[B33] Hargreaves, S.K., Williams, R.J., and Hofmockel, K.S. (2015) Environmental filtering of microbial communities in agricultural soil shifts with crop growth. PLoS One 10: e0134345. 26226508 10.1371/journal.pone.0134345 PMC4520589

[B34] Houlden, A., Timms-Wilson, T.M., Day, M.J., and Bailey, M.J. (2008) Influence of plant developmental stage on microbial community structure and activity in the rhizosphere of three field crops. FEMS Microbiol Ecol 65: 193–201. 18616582 10.1111/j.1574-6941.2008.00535.x

[B35] Hudz, S.O., and Skivka, L.M. (2021) Formation of the eubacterial complex in the rhyosphere of sugar beet (*Beta vulgaris*) under different fertilization systems. Biotechnol Acta 14: 81–86.

[B36] Huo, Y., Kang, J.P., Park, J.K., Li, J., Chen, L., and Yang, D.C. (2018) *Rhodanobacter ginsengiterrae* sp. nov., an antagonistic bacterium against root rot fungal pathogen *Fusarium solani*, isolated from ginseng rhizospheric soil. Arch Microbiol 200: 1457–1463. 30116848 10.1007/s00203-018-1560-9

[B89] Ikeda, S., Watanabe, K., Minamisawa, K., and Ytow, N. (2004) Evaluation of soil DNA from arable land in Japan using a modified direct-extraction method. Microbes Environ 19: 301–309.

[B37] Ikeda, S., Suzuki, K., Kawahara, M., Noshiro, M., and Takahashi, N. (2014) An assessment of urea-formaldehyde fertilizer on the diversity of bacterial communities in onion and sugar beet. Microbes Environ 29: 231–234. 24882062 10.1264/jsme2.ME13157 PMC4103532

[B39] Kloepper, J.W., Leong, J., Teintze, M., and Schroth, M.N. (1980) Enhanced plant growth by siderophores produced by plant growth-promoting rhizobacteria. Nature 286: 885–886.

[B40] Koga, N. (2008) An energy balance under a conventional crop rotation system in northern Japan: Perspectives on fuel ethanol production from sugar beet. Agric Ecosyst Environ 125: 101–110.

[B41] Kolton, M., Erlacher, A., Berg, G., and Cytryn, E. (2016) The *Flavobacterium* genus in the plant holobiont: ecological, physiological, and applicative insights. In *Microbial Models: from Environmental to Industrial Sustainability*. Castro-Sowinski, S. (ed.) Singapore: Springer, pp. 189–207.

[B42] Lugtenberg, B., and Kamilova, F. (2009) Plant-growth-promoting rhizobacteria. Annu Rev Microbiol 63: 541–556. 19575558 10.1146/annurev.micro.62.081307.162918

[B43] Luo, D., Langendries, S., Mendez, S.G., De Ryck, J., Liu, D., Beirinckx, S., et al. (2019) Plant growth promotion driven by a novel *Caulobacter* strain. Mol Plant Microbe Interact 32: 1162–1174. 30933667 10.1094/MPMI-12-18-0347-R

[B44] Magoc, T., and Salzberg, S.L. (2011) FLASH: fast length adjustment of short reads to improve genome assemblies. Bioinformatics 27: 2957–2963. 21903629 10.1093/bioinformatics/btr507 PMC3198573

[B45] Masuda, S., Bao, Z., Okubo, T., Sasaki, K., Ikeda, S., Shinoda, R., et al. (2016) Sulfur fertilization changes the community structure of rice root-, and soil-associated bacteria. Microbes Environ 31: 70–75. 26947443 10.1264/jsme2.ME15170 PMC4791119

[B46] Matsuhira, H., Kitazaki, K., Matsui, K., Kubota, K., Kuroda, Y., and Kubo, T. (2022) Selection of nuclear genotypes associated with the thermo-sensitivity of Owen-type cytoplasmic male sterility in sugar beet (*Beta vulgaris* L.). Theor Appl Genet 135: 1457–1466. 35147716 10.1007/s00122-022-04046-7

[B47] Mayerhofer, J., Thuerig, B., Oberhaensli, T., Enderle, E., Lutz, S., Ahrens, C.H., et al. (2021) Indicative bacterial communities and taxa of disease-suppressing and growth-promoting composts and their associations to the rhizoplane. FEMS Microbiol Ecol 97: fiab134. 34549287 10.1093/femsec/fiab134 PMC8478479

[B48] Mendes, R., Kruijt, M., De Bruijn, I., Dekkers, E., van der Voort, M., Schneider, J.H., et al. (2011) Deciphering the rhizosphere microbiome for disease-suppressive bacteria. Science 332: 1097–1100. 21551032 10.1126/science.1203980

[B49] Monteiro, R.A., Balsanelli, E., Wassem, R., Marin, A.M., Brusamarello-Santos, L.C., Schmidt, M.A., et al. (2012) *Herbaspirillum*-plant interactions: microscopical, histological and mole­cular aspects. Plant Soil 356: 175–196.

[B50] Morais, M.C., Mucha, Â., Ferreira, H., Gonçalves, B., Bacelar, E., and Marques, G. (2019) Comparative study of plant growth‐promoting bacteria on the physiology, growth and fruit quality of strawberry. J Sci Food Agric 99: 5341–5349. 31058322 10.1002/jsfa.9773

[B51] Nakagawa, Y. (2015) *Cytophagales*. In *Bergey’s Manual of Systematics of Archaea and Bacteria.* Hoboken, NJ: John Wiley & Sons, pp. 1–2.

[B52] Nakamura, Y., Ishibashi, M., Kamitani, Y., and Tsurumaru, H. (2020) Microbial community ana­lysis of digested liquids exhibiting different methane production potential in methane fermentation of swine feces. Appl Biochem Biotechnol 191: 1140–1154. 31965417 10.1007/s12010-020-03228-7

[B53] Naushad, S., Adeolu, M., Wong, S., Sohail, M., Schellhorn, H.E., and Gupta, R.S. (2015) A phylogenomic and mole­cular marker based taxonomic framework for the order *Xanthomonadales*: proposal to transfer the families *Algiphilaceae* and *Solimonadaceae* to the order *Nevskiales* ord. nov. and to create a new family within the order *Xanthomonadales*, the family *Rhodanobacteraceae* fam. nov., containing the genus *Rhodanobacter* and .... Antonie van Leeuwenhoek 107: 467–485. 25481407 10.1007/s10482-014-0344-8

[B54] O’Sullivan, D.J., and O’Gara, F. (1992) Traits of fluorescent *Pseudomonas* spp. involved in suppression of plant root pathogens. Microbiol Rev 56: 662–676. 1480114 10.1128/mr.56.4.662-676.1992 PMC372893

[B55] Okada, H., and Harada, H. (2007) Effects of tillage and fertilizer on nematode communities in a Japanese soybean field. Appl Soil Ecol 35: 582–598.

[B56] Okazaki, K., Iino, T., Kuroda, Y., Taguchi, K., Takahashi, H., Ohwada, T., et al. (2014) An assessment of the diversity of culturable bacteria from main root of sugar beet. Microbes Environ 29: 220–223. 24789987 10.1264/jsme2.ME13182 PMC4103529

[B57] Okazaki, K., Tsurumaru, H., Hashimoto, M., Takahashi, H., Okubo, T., Ohwada, T., et al. (2021) Community ana­lysis-based screening of plant growth-promoting bacteria for sugar beet. Microbes Environ 36: ME20137. 33907063 10.1264/jsme2.ME20137 PMC8209457

[B58] Pankratov, T.A., Kulichevskaya, I.S., Liesack, W., and Dedysh, S.N. (2006) Isolation of aerobic, gliding, xylanolytic and laminarinolytic bacteria from acidic *Sphagnum* peatlands and emended description of *Chitinophaga* *arvensicola* Kämpfer *et al.* 2006. Int J Syst Evol Microbiol 56: 2761–2764. 17158974 10.1099/ijs.0.64451-0

[B59] Pérez, J., Moraleda‐Muñoz, A., Marcos‐Torres, F.J., and Muñoz‐Dorado, J. (2016) Bacterial predation: 75 years and counting!. Environ Microbiol 18: 766–779. 26663201 10.1111/1462-2920.13171

[B60] Podosokorskaya, O.A., Bonch-Osmolovskaya, E.A., Novikov, A.A., Kolganova, T.V., and Kublanov, I.V. (2013) *Ornatilinea apprima* gen. nov., sp. nov., a cellulolytic representative of the class *Anaerolineae*. Int J Syst Evol Microbiol 63: 86–92. 22328612 10.1099/ijs.0.041012-0

[B61] Postma, J., Scheper, R.W.A., and Schilder, M.T. (2010) Effect of successive cauliflower plantings and *Rhizoctonia solani* AG 2-1 inoculations on disease suppressiveness of a suppressive and a conducive soil. Soil Biol Biochem 42: 804–812.

[B62] Raymaekers, K., Poneta, L., Holtappels, D., Berckmans, B., and Cammuea, B.P.A. (2020) Screening for novel biocontrol agents applicable in plant disease management—A review. Biol Control 144: 104240.

[B63] Reichenbach, H. (2015) *Myxococcales*. In *Bergey’s Manual of Systematics of Archaea and Bacteria*. Hoboken, NJ: John Wiley & Sons, pp. 1–31.

[B64] Sakuma, F., Maeda, M., Takahashi, M., Hashizume, K., and Kondo, N. (2011) Suppression of common scab of potato caused by *Streptomyces turgidiscabies* using lopsided oat green manure. Plant Dis 95: 1124–1130. 30732065 10.1094/PDIS-08-10-0615

[B65] Santiago, C.D., Yagi, S., Ijima, M., Nashimoto, T., Sawada, M., Ikeda, S., et al. (2017) Bacterial compatibility in combined inoculations enhances the growth of potato seedlings. Microbes Environ 32: 14–17. 28163278 10.1264/jsme2.ME16127 PMC5371070

[B66] Shi, Y., Lou, K., and Li, C. (2009) Promotion of plant growth by phytohormone-producing endophytic microbes of sugar beet. Biol Fertil Soils 45: 645–653.

[B67] Shi, Y., Yang, H., Zhang, T., Sun, J., and Lou, K. (2014) Illumina-based ana­lysis of endophytic bacterial diversity and space-time dynamics in sugar beet on the north slope of Tianshan mountain. Appl Microbiol Biotechnol 98: 6375–6385. 24752839 10.1007/s00253-014-5720-9

[B68] Shin, D.S., Park, M.S., Jung, S.R., Lee, M.S., Lee, K.H., Bae, K.S., et al. (2007) Plant growth-promoting potential of endophytic bacteria isolated from roots of coastal sand dune plants. J Microbiol Biotechnol 17: 1361–1368. 18051606

[B69] Steingrobe, B. (2001) Root renewal of sugar beet as a mechanism of P uptake efficiency. J Plant Nutr Soil Sci 164: 533–539.

[B70] Steingrobe, B. (2005) A sensitivity ana­lysis for assessing the relevance of fine‐root turnover for P and K uptake. J Plant Nutr Soil Sci 168: 496–502.

[B71] Thijs, S., Weyens, N., Sillen, W., Gkorezis, P., Carleer, R., and Vangronsveld, J. (2014) Potential for plant growth promotion by a consortium of stress‐tolerant 2, 4‐dinitrotoluene‐degrading bacteria: isolation and characterization of a military soil. Microb Biotechnol 7: 294–306. 24467368 10.1111/1751-7915.12111 PMC4241723

[B72] Toyota, K., and Watanabe, T. (2013) Recent trends in microbial inoculants in agriculture. Microbes Environ 28: 403–404. 24366038 10.1264/jsme2.ME2804rh PMC4070700

[B73] Tsurumaru, H., Okubo, T., Okazaki, K., Hashimoto, M., Kakizaki, K., Hanzawa, E., et al. (2015) Metagenomic ana­lysis of the bacterial community associated with the taproot of sugar beet. Microbes Environ 30: 63–69. 25740621 10.1264/jsme2.ME14109 PMC4356465

[B74] Unno, Y., Shinano, T., Minamisawa, K., and Ikeda, S. (2015) Bacterial community shifts associated with high abundance of *Rhizobium* spp. in potato roots under macronutrient-deficient conditions. Soil Biol Biochem 80: 232–236.

[B75] Vida, C., Bonilla, N., de Vicente, A., and Cazorla, F.M. (2016) Microbial profiling of a suppressiveness-induced agricultural soil amended with composted almond shells. Front Microbiol 7: 4. 26834725 10.3389/fmicb.2016.00004 PMC4722121

[B76] Vurukonda, S.S.K.P., Vardharajula, S., Shrivastava, M., and SkZ, A. (2016) Enhancement of drought stress tolerance in crops by plant growth promoting rhizobacteria. Microbiol Res 184: 13–24. 26856449 10.1016/j.micres.2015.12.003

[B77] Wang, G., Govinden, R., Chenia, H.Y., Ma, Y., Guo, D., and Ren, G. (2019) Suppression of *Phytophthora* blight of pepper by biochar amendment is associated with improved soil bacterial properties. Biol Fertil Soils 55: 813–824.

[B78] Wang, W., Luo, X., Ye, X., Chen, Y., Wang, H., Wang, L., et al. (2020) Predatory *Myxococcales* are widely distributed in and closely correlated with the bacterial community structure of agricultural land. Appl Soil Ecol 146: 103365.

[B79] Wu, G.D., Lewis, J.D., Hoffmann, C., Chen, Y.Y., Knight, R., Bittinger, K., et al. (2010) Sampling and pyrosequencing methods for characterizing bacterial communities in the human gut using 16S sequence tags. BMC Microbiol 10: 206. 20673359 10.1186/1471-2180-10-206 PMC2921404

[B80] Xie, C.H., and Yokota, A. (2006) *Sphingomonas azotifigens* sp. nov., a nitrogen-fixing bacterium isolated from the roots of *Oryza sativa*. Int J Syst Evol Microbiol 56: 889–893. 16585711 10.1099/ijs.0.64056-0

[B81] Yang, E., Sun, L., Ding, X., Sun, D., Liu, J., and Wang, W. (2019) Complete genome sequence of *Caulobacter flavus* RHGG3^T^, a type species of the genus *Caulobacter* with plant growth-promoting traits and heavy metal resistance. 3 Biotech 9: 42. 10.1007/s13205-019-1569-z PMC6330504 30675452

[B82] Yin, C., Hulbert, S.H., Schroeder, K.L., Mavrodi, O., Mavrodi, D., Dhingra, A., et al. (2013) Role of bacterial communities in the natural suppression of *Rhizoctonia solani* bare patch disease of wheat (*Triticum aestivum* L.). Appl Environ Microbiol 79: 7428–7438. 24056471 10.1128/AEM.01610-13 PMC3837727

[B83] Yin, C., Casa Vargas, J.M., Schlatter, D.C., Hagerty, C.H., Hulbert, S.H., and Paulitz, T.C. (2021) Rhizosphere community selection reveals bacteria associated with reduced root disease. Microbiome 9: 86. 33836842 10.1186/s40168-020-00997-5 PMC8035742

[B84] Zachow, C., Tilcher, R., and Berg, G. (2008) Sugar beet-associated bacterial and fungal communities show a high indigenous antagonistic potential against plant pathogens. Microb Ecol 55: 119–129. 18060449 10.1007/s00248-007-9257-7

[B85] Zachow, C., Müller, H., Tilcher, R., and Berg, G. (2014) Differences between the rhizosphere microbiome of *Beta vulgaris* ssp. *maritima*—ancestor of all beet crops—and modern sugar beets. Front Microbiol 5: 415. 25206350 10.3389/fmicb.2014.00415 PMC4144093

[B86] Zarraonaindia, I., Owens, S.M., Weisenhorn, P., West, K., Hampton-Marcell, J., Lax, S., et al. (2015) The soil microbiome influences grapevine-associated microbiota. mBio 6: e02527-14. 25805735 10.1128/mBio.02527-14 PMC4453523

[B87] Zhang, L., Gao, J.S., Kim, S.G., Zhang, C.W., Jiang, J.Q., Ma, X.T., et al. (2016) *Novosphingobium oryzae* sp. nov., a potential plant-promoting endophytic bacterium isolated from rice roots. Int J Syst Evol Microbiol 66: 302–307. 26514117 10.1099/ijsem.0.000718

[B88] Zhang, Z., Zhang, Q., Cui, H., Li, Y., Xu, N., Lu, T., et al. (2022) Composition identification and functional verification of bacterial community in disease‐suppressive soils by machine learning. Environ Microbiol 24: 3405–3419. 35049096 10.1111/1462-2920.15902

