# Supplementary material for: Seasonal Shifts in Bacterial Community Structures in the Lateral Root of Sugar Beet Grown in an Andosol Field in Japan
Source: Microbes Environ. 2023 Feb 9;38(1):ME22071. doi: 10.1264/jsme2.ME22071 (PMC10037095; doi:10.1264/jsme2.ME22071)
Supplement: Supplementary file 1 — Supplementary Material [file 38_22071_s1.pdf]

Supplementary materials

Title: Seasonal Shifts in Bacterial Community Structures in the Lateral Root of Sugar Beet Grown in an Andosol Field in Japan

Authors: Seishi Ikeda*[1†], Kazuyuki Okazaki[1†], Hiroyuki Takahashi[1], Hirohito Tsurumaru[2], and Kiwamu Minamisawa[3]

Author affiliation: [1]Memuro Research Station, Hokkaido Agricultural Research Center, National Agriculture and Food Research Organization, 9-4 Shinsei-minami, Memuro, Kasai-gun, Hokkaido 082-0081, Japan; [2]Faculty of Agriculture, Kagoshima University, 1-21-24, Korimoto, Kagoshima 890-0065, Japan; and [3]Graduate School of Life Science, Tohoku University, 2–1–1 Katahira, Aoba-ku, Sendai, Miyagi 980–8577, Japan

*__Corresponding author__: Seishi Ikeda, Memuro Research Station, Hokkaido Agricultural Research Center, National Agriculture and Food Research Organization, 9-4 Shinsei-minami, Memuro, Kasai-gun, Hokkaido 082-0081, Japan. E-mail: sikeda67@affrc.go.jp; Tel.: +81-155-62-9276; Fax: +81-155-61-2127.

† S.I. and K.O. equally contributed to this study.

Methods

BLASTN (Altschul et al., 1990) was used to conduct Blast search in order to identify the nearest relatives of ASVs in the GenBank database with the settings of excluding uncultured environmental sample sequences and otherwise default parameters.

References

Altschul S.F., Gish W., Miller W., Myers E.W., and Lipman D.J. (1990) Basic local alignment search tool. *J Mol Biol* **215**:403–410.

Table S1. Chemical characteristics of the soil sample on July 15, 2014 in the present study

pH (H_2O)	NH_4-N ($mg \cdot kg^{-1}$)	NO_3-N ($mg \cdot kg^{-1}$)	Total nitrogen ($g\ kg^{-1}$)	Available P_2O_5 ($mg \cdot kg^{-1}$)	Exchangeable cations			Phosphate absorption coefficient (mg/100g)	CEC ($cmolc \cdot kg^{-1}$)
					K_2O ($mg \cdot kg^{-1}$)	MgO ($mg \cdot kg^{-1}$)	CaO ($mg \cdot kg^{-1}$)		
6.0	5.2	17	2.3	214	451	478	2,294	1,331	176

Table S2. Relative abundaces (%) of lateral root-associated bacteria of sugar beet grown in an Andosol experimental field in Japan during the growth period from June to September at the levels from phylum to family

	Sampling month[b]			
Taxon[a]	Jun.	Jul.	Aug.	Sep.
Phylum				
Proteobacteria	50.3	54.3	56.1	58.7
Actinobacteria	25.3	16.7	16.1	10.5
Bacteroidetes	20.5	25.1	22.6	23.0
Acidobacteria	1.5	1.5	2.2	2.4
Verrucomicrobia	0.5	0.8	0.9	2.0
Chloroflexi	0.4	0.3	0.6	1.2
Others	1.5	1.3	1.4	2.1
Class				
Alphaproteobacteria	26.1	30.5	37.0	23.7
Actinobacteria	25.0	16.4	15.6	10.2
Betaproteobacteria	12.6	11.7	7.5	11.3
Saprospirae	12.0	16.8	18.0	17.8
Gammaproteobacteria	10.8	11.3	9.8	18.8
Sphingobacteriia	6.2	5.9	3.6	2.5
Flavobacteriia	1.3	1.4	0.4	0.9
Cytophagia	1.0	1.0	0.7	1.7
Deltaproteobacteria	0.9	0.8	1.7	4.8
Others	4.2	4.2	5.8	8.4
Order				
Actinomycetales	25.0	16.4	15.6	10.2
Saprospirales	12.0	16.8	18.0	17.8
Burkholderiales	11.7	11.0	6.9	10.0
Rhizobiales	11.6	13.8	15.2	10.6
Sphingomonadales	8.5	9.5	15.8	8.5
Sphingobacteriales	6.2	5.9	3.6	2.5
Xanthomonadales	5.5	7.0	8.7	13.1
Caulobacterales	5.5	6.0	4.2	2.1
Pseudomonadales	4.1	3.4	0.6	0.8
Flavobacteriales	1.3	1.4	0.4	0.9
Cytophagales	1.0	1.0	0.7	1.7
Myxococcales	0.8	0.6	1.5	4.5
Unclassified *Gammaproteobacteria* (c)	0.5	0.4	0.2	3.6
Ellin329	0.3	0.7	1.2	1.1
Others	6.1	6.1	7.4	12.6

	Sampling month			
Taxon	Jun.	Jul.	Aug.	Sep.
Family				
Streptomycetaceae	17.7	9.1	7.6	3.6
Chitinophagaceae	11.9	16.7	17.9	17.7
Oxalobacteraceae	8.5	5.1	1.7	0.9
Sphingomonadaceae	8.3	9.0	14.8	8.2
Sphingobacteriaceae	6.2	5.7	3.5	1.9
Caulobacteraceae	5.5	6.0	4.2	2.1
Xanthomonadaceae	4.9	5.5	6.3	4.1
Rhizobiaceae	4.6	5.0	5.9	3.6
Pseudomonadaceae	4.1	3.4	0.6	0.8
Nocardioidaceae	4.1	3.4	2.3	1.3
Hyphomicrobiaceae	2.7	2.8	2.6	2.1
Bradyrhizobiaceae	2.3	3.5	4.1	3.1
Comamonadaceae	2.1	4.8	4.0	8.2
Phyllobacteriaceae	1.7	2.1	1.9	0.9
Cytophagaceae	1.0	1.0	0.7	1.7
Burkholderiaceae	0.9	1.0	1.3	0.9
Flavobacteriaceae	0.9	1.1	0.2	0.4
Actinosynnemataceae	0.8	1.1	2.2	1.9
Sinobacteraceae	0.6	1.5	2.4	8.9
Gammaproteobacteria (c)	0.5	0.4	0.2	3.6
Ellin329 (o)	0.3	0.7	1.2	1.1
Haliangiaceae	0.2	0.2	0.4	1.4
Pseudonocardiaceae	0.2	0.4	1.1	1.3
Unclassified *Myxococcales* (o)	0.1	0.1	0.4	2.2
Others	10.0	10.2	12.6	18.2

[a]Taxon with 1% or more than 1% of relative abundance in any one of sampling months at the levels from phylum to family are shown. The relative abundance was caluculated based on 5491 reads per sample. A letter in parentheses (c and o) indicates the closest taxa (class and order, respectively) at each taxonomic level. Taxa highlited with gray are dominant taxa in all sampling months and taxa indicated in bold font showed no significant differences for the relative abundance among the sampling months.

[b]Results of average (n=6) are shown.

Table S3. Relative abundaces (%) of lateral root-associated bacteria of sugar beet grown in an Andosol experimental field in Japan during the growth period from June to September at the levels of genus and species

Genus[a]	Sampling month[b] Jun.	Jul.	Aug.	Sep.	Species[a]	Sampling month[b] Jun.	Jul.	Aug.	Sep.
Streptomyces	15.7	8.3	7.0	3.4	Unclassified *Streptomyces* (g)	15.3	8.0	6.7	3.0
Unclassified *Chitinophagaceae* (f)'	4.7	7.8	7.6	5.9	Unclassified *Chitinophagaceae* (f)	4.7	7.8	7.6	5.9
Pedobacter	4.6	3.2	1.8	0.6	Unclassified *Pedobacter* (g)	4.2	3.0	1.6	0.6
Pseudomonas	4.1	3.4	0.6	0.8	Unclassified *Pseudomonas* (g)	3.9	3.1	0.6	0.8
Janthinobacterium	3.9	1.5	0.3	0.2	**Unclassified *Niastella* (g)**	3.7	4.7	4.5	5.3
Niastella	3.7	4.7	4.5	5.3	Unclassified *Kribbella* (g)	3.6	3.0	2.0	1.2
Caulobacter	3.6	3.9	2.5	1.3	*Caulobacter henricii*	3.6	3.6	2.3	1.0
Kribbella	3.6	3.0	2.0	1.2	Unclassified *Janthinobacterium* (g)	3.0	1.4	0.2	0.2
Rhizobium	3.0	2.7	3.5	1.6	Unclassified *Oxalobacteraceae* (f)	2.8	2.5	0.9	0.6
Unclassified *Oxalobacteraceae* (f)	2.8	2.5	0.9	0.6	Unclassified *Rhizobium* (g)	2.6	2.3	3.3	1.6
Chitinophaga	2.6	2.5	3.8	3.9	Unclassified *Sphingobium* (g)	2.3	1.2	1.2	0.4
Rhodanobacter	2.5	2.2	1.9	0.9	Unclassified *Devosia* (g)	2.2	2.4	1.7	1.5
Sphingobium	2.3	1.3	1.2	0.5	Unclassified *Streptomycetaceae* (f)	2.0	0.8	0.6	0.2
Devosia	2.2	2.4	1.7	1.5	*Chitinophaga arvensicola*	1.9	1.2	2.4	1.0
Novosphingobium	2.0	2.5	7.1	4.4	**Unclassified *Rhodanobacter* (g)**	1.9	1.7	1.7	0.8
Unclassified *Streptomycetaceae* (f)	2.0	0.8	0.6	0.2	Unclassified *Comamonadaceae* (f)	1.7	3.5	3.0	6.2
Sphingomonas	1.9	3.8	4.4	2.7	**Unclassified *Sphingobacteriaceae* (f)**	1.3	1.9	1.4	0.9
Unclassified *Comamonadaceae* (f)	1.7	3.5	3.0	6.2	Unclassified *Bradyrhizobiaceae* (f)	1.3	1.3	1.3	0.4
Sphingopyxis	1.4	0.9	0.9	0.1	Unclassified *Mesorhizobium* (g)	1.3	1.6	1.5	0.7
**Unclassified *Sphingobacteriaceae* (f)**	1.3	1.9	1.4	0.9	Unclassified *Sphingomonas* (g)	1.2	1.9	3.4	1.7
Unclassified *Bradyrhizobiaceae* (f)	1.3	1.3	1.3	0.4	*Asticcacaulis biprosthecium*	1.1	1.1	0.8	0.1
Mesorhizobium	1.3	1.6	1.5	0.7	Unclassified *Novosphingobium* (g)	1.1	1.4	3.7	3.5
Asticcacaulis	1.1	1.1	0.8	0.1	Unclassified *Rhizobiaceae* (f)	1.1	1.0	0.6	0.7
**Unclassified *Rhizobiaceae* (f)**	1.1	1.0	0.6	0.7	Unclassified *Novosphingobium* (g)	0.9	1.0	3.4	0.9
Burkholderia	0.9	1.0	1.3	0.9	Unclassified *Bradyrhizobium* (g)	0.9	1.8	2.3	2.2
Bradyrhizobium	0.9	2.0	2.7	2.5	Unclassified *Cytophagaceae* (f)	0.8	0.7	0.4	1.2
Flavobacterium	0.9	1.1	0.2	0.4	**Unclassified *Lysobacter* (g)**	0.8	1.1	0.8	0.5
Lysobacter	0.8	1.2	1.0	0.7	Unclassified *Chitinophagaceae* (f)	0.7	1.6	1.8	2.5
**Unclassified *Cytophagaceae* (f)**	0.8	0.7	0.4	1.2	*Sphingomonas azotifigens*	0.7	1.6	0.9	0.9
Unclassified *Chitinophagaceae* (f)"	0.7	1.6	1.8	2.5	Unclassified *Chitinophaga* (g)	0.7	1.3	1.4	2.8
Agrobacterium	0.5	1.2	1.8	1.2	Unclassified *Agrobacterium* (g)	0.5	1.2	1.8	1.2
Unclassified *Gammaproteobacteria* (c)	0.5	0.4	0.2	3.6	Unclassified *Gammaproteobacteria* (c)	0.5	0.4	0.2	3.6
Steroidobacter	0.3	0.7	1.6	5.9	Unclassified *Steroidobacter* (g)	0.3	0.7	1.6	5.9
Unclassified *Sinobacteraceae* (f)	0.3	0.7	0.8	3.0	Unclassified *Sinobacteraceae* (f)	0.3	0.7	0.8	3.0
Dokdonella	0.3	0.9	1.6	1.5	Unclassified *Dokdonella* (g)	0.3	0.9	1.6	1.5
Unclassified *Ellin329* (o)	0.3	0.7	1.2	1.1	Unclassified *Ellin329* (o)	0.3	0.7	1.2	1.1
**Unclassified *Haliangiaceae* (f)**	0.2	0.2	0.4	1.4	Unclassified *Haliangiaceae* (f)	0.2	0.2	0.4	1.4
Kutzneria	0.2	0.3	1.2	0.8	Unclassified *Kutzneria* (g)	0.2	0.3	1.2	0.8
Amycolatopsis	0.2	0.4	1.1	1.3	Unclassified *Amycolatopsis* (g)	0.2	0.4	1.1	1.3
Unclassified *Myxococcales* (o)	0.1	0.1	0.4	2.2	Unclassified *Myxococcales* (o)	0.1	0.1	0.4	2.2
Others	17.6	18.8	21.4	25.8	Others	23.6	24.3	24.9	28.3

[a]Taxon with 1% or more than 1% of relative abundance in any one of sampling months at the genus and species levels are shown. The relative abundance was caluculated based on 5491 reads per sample. Letters in parentheses (c, f, g, and o) indicate the closest taxa (class, family, genus and order, respectively) at each taxonomic level. Taxa highlited with gray are dominant taxa in all sampling months and taxa indicated in bold font showed no significant differences for the relative abundance among the sampling months.

[b]Results of average (n=6) are shown.

Table S4. ASVs with 0.1% or more than 0.1% of relative abundance in any one of sampling months for lateral root-associated bacteria of sugar beet grown in an Andosol experimental field in Japan during the growth period from June to September

Close taxon[a] /	Sampling month[c]									
ASV ID[b]	June		July		August		September		Sig.[d]	Note
Unclassified *Kutzneria* (1 ASV)										
**ASV_034**	0.17 ± 0.17	b	0.33 ± 0.19	b	1.20 ± 0.35	a	0.79 ± 0.76	ab	**	*Actinobacteria*
Kribbella (4 ASVs)										
**ASV_012**	1.77 ± 0.72	a	1.53 ± 0.45	a	1.15 ± 0.46	ab	0.74 ± 0.38	b	**	*Actinobacteria*
**ASV_038**	1.38 ± 0.30	a	0.98 ± 0.48	ab	0.47 ± 0.18	bc	0.25 ± 0.26	c	***	*Actinobacteria*
ASV_115	0.24 ± 0.37		0.11 ± 0.18		0.24 ± 0.12		0.14 ± 0.32			*Actinobacteria*
ASV_134	0.22 ± 0.23	ab	0.33 ± 0.19	a	0.11 ± 0.09	ab	0.03 ± 0.08	b	*	*Actinobacteria*
Unclassified *Amycolatopsis* (1 ASV)										
**ASV_017**	0.16 ± 0.12	b	0.39 ± 0.18	b	1.09 ± 0.24	a	1.25 ± 0.52	a	***	*Actinobacteria*
Unclassified *Streptomycetaceae* (7 ASVs)										
**ASV_064**	1.18 ± 0.43	a	0.53 ± 0.29	ab	0.23 ± 0.21	b	0.12 ± 0.18	b	***	*Actinobacteria*
ASV_184	0.62 ± 0.82	a	0.10 ± 0.17	ab	0.003 ± 0.007	b	0 ± 0	b	**	*Actinobacteria*
ASV_165	0.19 ± 0.34		0.11 ± 0.09		0.16 ± 0.12		0.05 ± 0.13			*Actinobacteria*
ASV_159	0 ± 0	c	0.03 ± 0.05	bc	0.20 ± 0.05	a	0.07 ± 0.07	b	***	*Actinobacteria*
Streptomyces in *Streptomycetaceae* (6 ASVs)										
**ASV_001**	13.87 ± 5.06	a	7.19 ± 2.69	b	5.99 ± 1.15	b	2.60 ± 0.99	c	***	*Actinobacteria*
**ASV_039**	1.20 ± 0.32	a	0.67 ± 0.23	b	0.59 ± 0.26	b	0.37 ± 0.15	b	***	*Actinobacteria*
ASV_372	0.21 ± 0.51		0.08 ± 0.13		0 ± 0		0 ± 0			*Actinobacteria*
Unclassified *Chitinophagaceae* (147 ASVs)										
**ASV_002**	1.21 ± 0.41	b	3.41 ± 1.10	a	3.45 ± 1.99	a	2.01 ± 1.05	ab	**	*Bacteroidetes*
ASV_044	0.73 ± 0.38		0.79 ± 0.31		0.48 ± 0.17		0.44 ± 0.28			*Bacteroidetes*
ASV_071	0.66 ± 0.52	a	0.44 ± 0.23	a	0.28 ± 0.35	ab	0.03 ± 0.07	b	*	*Bacteroidetes*
ASV_144	0.62 ± 0.52	a	0.11 ± 0.06	b	0.08 ± 0.07	b	0.00 ± 0.01	b	***	*Bacteroidetes*
ASV_092	0.29 ± 0.20		0.28 ± 0.18		0.36 ± 0.11		0.12 ± 0.14			*Bacteroidetes*
ASV_050	0.27 ± 0.36		0.56 ± 0.39		0.69 ± 0.32		0.36 ± 0.22			*Bacteroidetes*
ASV_073	0.24 ± 0.10		0.37 ± 0.14		0.30 ± 0.10		0.38 ± 0.13			*Bacteroidetes*
ASV_068	0.16 ± 0.12	b	0.37 ± 0.24	ab	0.29 ± 0.05	ab	0.47 ± 0.10	a	**	*Bacteroidetes*
ASV_190	0.15 ± 0.08		0.13 ± 0.07		0.09 ± 0.06		0.02 ± 0.03			*Bacteroidetes*
ASV_205	0.12 ± 0.17		0.10 ± 0.10		0.07 ± 0.06		0.01 ± 0.03			*Bacteroidetes*
ASV_070	0.11 ± 0.17	b	0.49 ± 0.19	a	0.34 ± 0.22	a	0.42 ± 0.20	a	**	*Bacteroidetes*
ASV_194	0.06 ± 0.09		0.23 ± 0.19		0.04 ± 0.08		0.05 ± 0.08			*Bacteroidetes*
ASV_042	0.05 ± 0.09	b	0.37 ± 0.25	a	0.70 ± 0.30	a	0.53 ± 0.32	a	***	*Bacteroidetes*
ASV_241	0.03 ± 0.06		0.12 ± 0.14		0.003 ± 0.007		0.07 ± 0.09			*Bacteroidetes*
ASV_052	0 ± 0	c	0.24 ± 0.10	b	0.45 ± 0.18	b	0.96 ± 0.45	a	***	*Bacteroidetes*
ASV_161	0 ± 0	c	0.04 ± 0.05	bc	0.09 ± 0.07	b	0.24 ± 0.06	a	***	*Bacteroidetes*
ASV_099	0 ± 0	b	0.05 ± 0.11	b	0.47 ± 0.14	a	0.16 ± 0.31	b	***	*Bacteroidetes*
ASV_164	0 ± 0	b	0.12 ± 0.13	ab	0.14 ± 0.08	a	0.06 ± 0.07	ab	*	*Bacteroidetes*
ASV_183	0 ± 0	b	0.06 ± 0.10	ab	0.10 ± 0.07	a	0.16 ± 0.14	a	***	*Bacteroidetes*
ASV_185	0 ± 0	b	0.03 ± 0.04	b	0.02 ± 0.02	b	0.22 ± 0.16	a	**	*Bacteroidetes*
ASV_363	0 ± 0		0.10 ± 0.16		0 ± 0		0.03 ± 0.08			*Bacteroidetes*
Chitinophaga in *Chitinophagaceae* (13 ASVs)										
**ASV_005**	1.90 ± 0.72	ab	1.24 ± 0.70	ab	2.38 ± 1.24	a	1.04 ± 0.48	b	*	*Bacteroidetes* (*C. arvensicola*)
ASV_049	0.28 ± 0.16		0.60 ± 0.24		0.49 ± 0.21		0.63 ± 0.39			*Bacteroidetes*
**ASV_021**	0.16 ± 0.29	b	0.35 ± 0.19	ab	0.75 ± 0.26	ab	1.97 ± 3.04	a	*	*Bacteroidetes*
ASV_237	0.10 ± 0.25		0.05 ± 0.08		0.06 ± 0.11		0.03 ± 0.07			*Bacteroidetes*
ASV_255	0 ± 0		0.07 ± 0.10		0.03 ± 0.05		0.12 ± 0.18			*Bacteroidetes*

Table S4. continued.

Close taxon[a] / ASV ID[b]	Sampling month[c] June		July		August		September		Sig.[d]	Note
Niastella in *Chitinophagaceae* (13 ASVs)										
ASV_008	1.85 ± 0.94		1.45 ± 0.50		0.89 ± 0.39		2.03 ± 1.00			*Bacteroidetes*
ASV_033	1.03 ± 0.26		0.82 ± 0.47		0.77 ± 0.18		0.51 ± 0.13			*Bacteroidetes*
ASV_061	0.30 ± 0.25		0.50 ± 0.31		0.38 ± 0.16		0.39 ± 0.11			*Bacteroidetes*
ASV_028	0.24 ± 0.38	b	0.61 ± 0.32	a	0.83 ± 0.26	a	0.95 ± 0.34	a	**	*Bacteroidetes*
ASV_045	0.21 ± 0.18		0.52 ± 0.41		0.63 ± 0.32		0.60 ± 0.31			*Bacteroidetes*
ASV_037	0.08 ± 0.10	b	0.56 ± 0.17	a	0.73 ± 0.43	a	0.74 ± 0.27	a	***	*Bacteroidetes*
ASV_152	0 ± 0	b	0.08 ± 0.10	ab	0.12 ± 0.07	a	0.13 ± 0.08	a	**	*Bacteroidetes*
ASV_251	0 ± 0	b	0.04 ± 0.09	ab	0.12 ± 0.13	a	0 ± 0	b	*	*Bacteroidetes*
Cytophagaceae (56 ASVs)										
ASV_085	0.14 ± 0.13	b	0.34 ± 0.15	a	0.19 ± 0.06	ab	0.43 ± 0.13	a	**	*Bacteroidetes*
ASV_145	0.12 ± 0.09		0.13 ± 0.05		0.09 ± 0.05		0.20 ± 0.10			*Bacteroidetes*
ASV_095	0.07 ± 0.12	b	0.06 ± 0.12	b	0.19 ± 0.12	ab	0.35 ± 0.35	a	*	*Bacteroidetes* (*Dyadobacter*)
ASV_234	0.04 ± 0.05		0.13 ± 0.11		0.04 ± 0.07		0.03 ± 0.07			*Bacteroidetes* (*Dyadobacter*)
ASV_199	0.01 ± 0.02	b	0.02 ± 0.02	b	0.02 ± 0.03	b	0.22 ± 0.14	a	**	*Bacteroidetes*
Flavobacterium (32 ASVs)										
ASV_175	0.25 ± 0.36	ab	0.30 ± 0.24	a	0.02 ± 0.02	b	0 ± 0	b	*	*Bacteroidetes*
ASV_204	0.18 ± 0.28		0.21 ± 0.20		0.05 ± 0.10		0.01 ± 0.02			*Bacteroidetes* (*F. succinicans*)
ASV_352	0.17 ± 0.42		0 ± 0		0.04 ± 0.10		0 ± 0			*Bacteroidetes* (*F. succinicans*)
ASV_126	0.05 ± 0.13		0.11 ± 0.13		0 ± 0		0.24 ± 0.55			*Bacteroidetes* (*F. succinicans*)
ASV_380	0 ± 0	b	0.12 ± 0.17	a	0.01 ± 0.01	ab	0 ± 0	b	*	*Bacteroidetes*
Pedobacter (13 ASVs)										
**ASV_014**	2.83 ± 1.07	a	1.68 ± 0.89	ab	0.79 ± 0.52	bc	0.24 ± 0.41	c	***	*Bacteroidetes*
ASV_051	0.61 ± 0.22		0.56 ± 0.31		0.63 ± 0.43		0.19 ± 0.09			*Bacteroidetes*
ASV_157	0.44 ± 0.38	a	0.17 ± 0.11	ab	0.08 ± 0.14	ab	0.05 ± 0.13	b	*	*Bacteroidetes*
ASV_153	0.32 ± 0.39		0.15 ± 0.18		0.12 ± 0.14		0 ± 0			*Bacteroidetes* (*P. terricola*)
ASV_104	0.24 ± 0.42		0.54 ± 0.72		0.09 ± 0.10		0.08 ± 0.13			*Bacteroidetes*
Caulobacteraceae (6 ASVs)										
**ASV_032**	1.09 ± 0.25	a	1.11 ± 0.43	a	0.81 ± 0.44	a	0.13 ± 0.16	b	***	*Alphaproteobacteria* (*A. biprosthecium*)
**ASV_003**	3.36 ± 0.64	a	3.52 ± 1.14	a	2.31 ± 0.72	a	1.02 ± 0.36	b	***	*Alphaproteobacteria* (*C. henricii*)
ASV_207	0.22 ± 0.24		0.08 ± 0.09		0.04 ± 0.05		0 ± 0			*Alphaproteobacteria* (*C. henricii*)
ASV_127	0.07 ± 0.07	b	0.27 ± 0.14	a	0.14 ± 0.09	ab	0.24 ± 0.11	a	**	*Alphaproteobacteria* (*Caulobacter*)
Unclassified *Ellin329* (23 ASVs)										
ASV_081	0.06 ± 0.10	b	0.28 ± 0.19	a	0.26 ± 0.08	a	0.47 ± 0.16	a	***	*Alphaproteobacteria*
ASV_215	0.03 ± 0.08		0.08 ± 0.10		0.06 ± 0.07		0.12 ± 0.17			*Alphaproteobacteria*
ASV_122	0 ± 0	b	0.08 ± 0.13	b	0.41 ± 0.37	a	0.02 ± 0.04	b	***	*Alphaproteobacteria*
ASV_148	0 ± 0	b	0.18 ± 0.10	a	0.10 ± 0.06	a	0.14 ± 0.13	a	**	*Alphaproteobacteria*
ASV_286	0 ± 0		0 ± 0		0.15 ± 0.33		0.05 ± 0.12			*Alphaproteobacteria*
ASV_287	0 ± 0		0 ± 0		0.03 ± 0.07		0.14 ± 0.17			*Alphaproteobacteria*
Unclassified *Bradyrhizobiaceae* (9 ASVs)										
ASV_056	0.84 ± 0.25	a	0.49 ± 0.13	b	0.62 ± 0.17	ab	0.12 ± 0.09	c	***	*Alphaproteobacteria*
ASV_112	0 ± 0	b	0.19 ± 0.22	ab	0.29 ± 0.10	a	0.07 ± 0.11	b	**	*Alphaproteobacteria*
ASV_135	0.27 ± 0.18	a	0.27 ± 0.18	a	0.12 ± 0.06	ab	0.02 ± 0.04	b	*	*Alphaproteobacteria*
ASV_149	0.22 ± 0.40		0.09 ± 0.11		0.12 ± 0.10		0.05 ± 0.07			*Alphaproteobacteria*
ASV_225	0 ± 0	b	0.17 ± 0.08	a	0.06 ± 0.05	ab	0.05 ± 0.11	b	***	*Alphaproteobacteria*
Bradyrhizobium in *Bradyrhizobiaceae* (2 ASVs)										
**ASV_004**	0.91 ± 0.35	b	1.81 ± 0.48	a	2.28 ± 0.41	a	2.23 ± 0.69	a	***	*Alphaproteobacteria*
ASV_090	0 ± 0	c	0.18 ± 0.09	b	0.39 ± 0.10	a	0.32 ± 0.17	ab	***	*Alphaproteobacteria*

Table S4. continued.

Close taxon[a] / ASV ID[b]	Sampling month[c] June	July	August	September	Sig.[d]	Note
Unclassified *Devosia* (12 ASVs)						
ASV_018	0.76 ± 0.22	1.07 ± 0.43	0.98 ± 0.20	0.93 ± 0.39		*Alphaproteobacteria*
ASV_114	0.59 ± 0.36 a	0.35 ± 0.10 ab	0.16 ± 0.05 b	0.02 ± 0.02 c	***	*Alphaproteobacteria*
ASV_096	0.51 ± 0.22 a	0.33 ± 0.23 ab	0.27 ± 0.37 ab	0.08 ± 0.07 b	*	*Alphaproteobacteria*
ASV_181	0.24 ± 0.15 a	0.05 ± 0.05 ab	0.12 ± 0.12 ab	0.01 ± 0.01 b	**	*Alphaproteobacteria*
ASV_151	0.13 ± 0.13	0.26 ± 0.21	0.08 ± 0.02	0.15 ± 0.06		*Alphaproteobacteria*
ASV_195	0 ± 0 b	0.27 ± 0.12 a	0.08 ± 0.11 b	0.09 ± 0.11 b	***	*Alphaproteobacteria*
Phyllobacteriaceae (6 ASVs)						
ASV_077	0.46 ± 0.16 a	0.45 ± 0.12 a	0.34 ± 0.11 a	0.15 ± 0.08 b	***	*Alphaproteobacteria* (*Aminobacter*)
ASV_029	0.73 ± 0.09 a	0.82 ± 0.24 a	0.98 ± 0.28 a	0.36 ± 0.09 b	***	*Alphaproteobacteria* (*Mesorhizobium*)
ASV_074	0.33 ± 0.07 ab	0.46 ± 0.06 a	0.36 ± 0.13 ab	0.28 ± 0.08 b	*	*Alphaproteobacteria* (*Mesorhizobium*)
ASV_121	0.20 ± 0.17 ab	0.30 ± 0.21 a	0.18 ± 0.11 ab	0.05 ± 0.07 b	*	*Alphaproteobacteria* (*Mesorhizobium*)
Rhizobiaceae (9 ASVs)						
**ASV_009**	0.42 ± 0.08 b	1.23 ± 0.41 a	1.77 ± 0.96 a	1.11 ± 0.42 a	***	*Alphaproteobacteria* (*Agrobacterium*)
**ASV_006**	1.75 ± 0.62 ab	1.52 ± 0.43 ab	2.19 ± 0.42 a	1.17 ± 0.29 b	**	*Alphaproteobacteria* (*Rhizobium*)
ASV_035	0.49 ± 0.43 ab	0.78 ± 0.36 ab	0.93 ± 0.27 a	0.23 ± 0.19 b	*	*Alphaproteobacteria* (*Rhizobium*)
ASV_097	0.46 ± 0.25 a	0.42 ± 0.20 a	0.21 ± 0.08 ab	0.06 ± 0.12 b	**	*Alphaproteobacteria* (*Rhizobium*)
ASV_119	0.31 ± 0.59	0 ± 0	0.19 ± 0.39	0.18 ± 0.20		*Alphaproteobacteria* (*Rhizobium*)
Unvlassified *Novosphingobium* (13 ASVs)						
**ASV_023**	0.85 ± 0.55 ab	0.49 ± 0.34 ab	1.35 ± 0.91 a	0.21 ± 0.33 b	**	*Alphaproteobacteria*
ASV_060	0.33 ± 0.56	0.20 ± 0.23	0.42 ± 0.47	0.65 ± 0.34		*Alphaproteobacteria*
**ASV_010**	0.20 ± 0.31 b	0.79 ± 0.60 ab	1.35 ± 0.52 a	1.78 ± 0.81 a	***	*Alphaproteobacteria*
ASV_202	0.15 ± 0.23	0.06 ± 0.12	0.09 ± 0.11	0.08 ± 0.20		*Alphaproteobacteria*
ASV_066	0.13 ± 0.15	0.04 ± 0.10	0.76 ± 1.16	0.13 ± 0.16		*Alphaproteobacteria*
ASV_288	0.12 ± 0.18	0.02 ± 0.05	0.06 ± 0.10	0 ± 0		*Alphaproteobacteria*
ASV_076	0.08 ± 0.20 b	0 ± 0 b	0.26 ± 0.49 ab	0.54 ± 0.38 a	**	*Alphaproteobacteria*
ASV_120	0.08 ± 0.14	0.05 ± 0.13	0.29 ± 0.46	0 ± 0		*Alphaproteobacteria*
**ASV_013**	0.07 ± 0.16 c	0.50 ± 0.43 bc	1.85 ± 1.28 a	0.56 ± 0.47 b	***	*Alphaproteobacteria*
ASV_169	0.03 ± 0.07	0.06 ± 0.06	0.15 ± 0.09	0.07 ± 0.08		*Alphaproteobacteria*
ASV_080	0 ± 0 c	0.22 ± 0.08 b	0.44 ± 0.12 a	0.33 ± 0.18 ab	***	*Alphaproteobacteria*
Unclassified *Sphingobium* (7 ASVs)						
**ASV_015**	2.03 ± 0.87 a	1.13 ± 0.43 a	1.16 ± 0.60 a	0.43 ± 0.15 b	***	*Alphaproteobacteria*
ASV_249	0.15 ± 0.10 a	0.08 ± 0.09 ab	0.03 ± 0.03 ab	0.02 ± 0.02 b	*	*Alphaproteobacteria*
ASV_395	0.13 ± 0.20	0.03 ± 0.07	0 ± 0	0 ± 0		*Alphaproteobacteria*
Sphingomonas (20 ASVs)						
**ASV_007**	1.19 ± 0.49 b	1.07 ± 0.34 b	2.49 ± 0.81 a	0.93 ± 0.85 b	**	*Alphaproteobacteria*
**ASV_016**	0.68 ± 0.17 b	1.58 ± 0.41 a	0.86 ± 0.42 b	0.94 ± 0.50 ab	**	*Alphaproteobacteria* (*S. azotifigens*)
ASV_036	0 ± 0 b	0.80 ± 0.24 a	0.85 ± 0.34 a	0.72 ± 0.25 a	***	*Alphaproteobacteria*
ASV_224	0 ± 0	0.13 ± 0.18	0.09 ± 0.11	0.02 ± 0.04		*Alphaproteobacteria*
ASV_335	0 ± 0	0.13 ± 0.15	0.01 ± 0.01	0.02 ± 0.04		*Alphaproteobacteria*
Sphingopyxis (5 ASVs)						
ASV_069	0.77 ± 0.78 a	0.44 ± 0.29 ab	0.34 ± 0.23 ab	0.03 ± 0.05 b	*	*Alphaproteobacteria*
ASV_086	0.44 ± 0.44	0.34 ± 0.28	0.27 ± 0.18	0.11 ± 0.08		*Alphaproteobacteria* (*S. alaskensis*)
ASV_137	0.14 ± 0.23	0.16 ± 0.29	0.23 ± 0.14	0 ± 0		*Alphaproteobacteria*

Table S4. continued.

Close taxon[a] / ASV ID[b]	June		July		August		September		Sig.[d]	Note
	Sampling month[c]									
Uncalssified *Comamonadaceae* (24 ASVs)										
ASV_059	0.94 ± 0.45	a	0.86 ± 0.29	a	0.19 ± 0.21	b	0.02 ± 0.06	b	***	*Betaproteobacteria*
ASV_041	0.29 ± 0.38		0.53 ± 0.23		0.99 ± 1.06		0.36 ± 0.09			*Betaproteobacteria*
ASV_091	0.12 ± 0.18	b	0.34 ± 0.17	ab	0.15 ± 0.14	ab	0.42 ± 0.28	a	*	*Betaproteobacteria*
**ASV_040**	0.10 ± 0.24	c	0.15 ± 0.18	bc	0.36 ± 0.18	b	1.34 ± 0.56	a	***	*Betaproteobacteria*
**ASV_025**	0.08 ± 0.16	c	0.48 ± 0.16	b	0.50 ± 0.33	b	2.13 ± 0.46	a	***	*Betaproteobacteria*
ASV_094	0.06 ± 0.07	c	0.56 ± 0.20	a	0.11 ± 0.05	bc	0.28 ± 0.12	ab	***	*Betaproteobacteria*
ASV_084	0 ± 0	c	0.06 ± 0.11	bc	0.33 ± 0.23	a	0.26 ± 0.31	ab	**	*Betaproteobacteria*
ASV_125	0 ± 0	c	0.02 ± 0.04	bc	0.06 ± 0.04	b	0.42 ± 0.25	a	***	*Betaproteobacteria*
ASV_132	0 ± 0		0.06 ± 0.09		0.18 ± 0.17		0.19 ± 0.21			*Betaproteobacteria*
ASV_143	0 ± 0		0.25 ± 0.42		0.02 ± 0.06		0.21 ± 0.23			*Betaproteobacteria*
ASV_150	0 ± 0	c	0.08 ± 0.08	b	0.09 ± 0.07	ab	0.19 ± 0.07	a	***	*Betaproteobacteria*
ASV_321	0 ± 0	b	0.01 ± 0.01	b	0.02 ± 0.02	b	0.10 ± 0.05	a	***	*Betaproteobacteria*
Unclassified *Oxalobacteraceae* (14 ASVs)										
**ASV_022**	1.85 ± 0.74	a	1.48 ± 0.54	a	0.70 ± 0.21	b	0.34 ± 0.19	b	***	*Betaproteobacteria*
ASV_128	0.59 ± 0.32	a	0.27 ± 0.09	b	0.10 ± 0.05	c	0 ± 0	d	***	*Betaproteobacteria*
ASV_113	0.46 ± 0.44	a	0.40 ± 0.08	a	0.11 ± 0.09	ab	0.03 ± 0.08	b	**	*Betaproteobacteria*
ASV_172	0.29 ± 0.16	a	0.12 ± 0.08	b	0.16 ± 0.10	ab	0.00 ± 0.01	c	***	*Betaproteobacteria*
ASV_179	0.22 ± 0.13		0.25 ± 0.17		0.04 ± 0.05		0.06 ± 0.05			*Betaproteobacteria*
ASV_367	0.15 ± 0.27		0.05 ± 0.13		0 ± 0		0 ± 0			*Betaproteobacteria*
ASV_212	0.02 ± 0.04	b	0.09 ± 0.09	ab	0.06 ± 0.04	ab	0.12 ± 0.06	a	*	*Betaproteobacteria*
ASV_253	0 ± 0		0.17 ± 0.37		0.01 ± 0.02		0.01 ± 0.02			*Betaproteobacteria*
Janthinobacterium in *Oxalobacteraceae* (5 ASVs)										
**ASV_030**	3.00 ± 1.39	a	1.28 ± 0.55	b	0.21 ± 0.09	c	0.18 ± 0.11	c	***	*Betaproteobacteria*
ASV_141	0.87 ± 0.48	a	0.15 ± 0.16	b	0.03 ± 0.03	b	0 ± 0	b	***	*Betaproteobacteria* (*J. lividum*)
Unclassified *Myxococcales* (94 ASVs)										
ASV_213	0.17 ± 0.18		0.04 ± 0.05		0.09 ± 0.08		0.03 ± 0.03			*Deltaproteobacteria*
ASV_244	0.13 ± 0.13		0.03 ± 0.04		0.04 ± 0.04		0.02 ± 0.02			*Deltaproteobacteria*
ASV_079	0.06 ± 0.10	c	0.13 ± 0.06	bc	0.31 ± 0.10	ab	0.46 ± 0.25	a	***	*Deltaproteobacteria*
ASV_093	0 ± 0	c	0 ± 0	c	0.18 ± 0.10	b	0.59 ± 0.31	a	***	*Deltaproteobacteria*
ASV_162	0 ± 0	c	0 ± 0	c	0.04 ± 0.03	b	0.30 ± 0.18	a	***	*Deltaproteobacteria*
ASV_210	0 ± 0	b	0 ± 0	b	0 ± 0	b	0.28 ± 0.13	a	***	*Deltaproteobacteria*
ASV_221	0 ± 0	b	0 ± 0	b	0.003 ± 0.007	b	0.22 ± 0.20	a	***	*Deltaproteobacteria*
ASV_261	0 ± 0	b	0 ± 0	b	0.01 ± 0.02	b	0.22 ± 0.15	a	***	*Deltaproteobacteria*
Unclassified *Haliangiaceae* in *Myxococcales* (33 ASVs)										
ASV_109	0 ± 0	b	0 ± 0	b	0.13 ± 0.12	ab	0.45 ± 0.34	a	***	*Deltaproteobacteria*
ASV_156	0 ± 0	b	0.07 ± 0.07	b	0.05 ± 0.05	b	0.29 ± 0.26	a	***	*Deltaproteobacteria*
ASV_170	0 ± 0	b	0 ± 0	b	0 ± 0	b	0.38 ± 0.43	a	***	*Deltaproteobacteria*
Unclassified *Gammaproteobacteria* (32 ASVs)										
ASV_310	0.24 ± 0.59		0.04 ± 0.07		0.01 ± 0.02		0 ± 0			*Gammaproteobacteria*
**ASV_054**	0.16 ± 0.40	b	0.14 ± 0.16	b	0.03 ± 0.05	b	1.85 ± 1.71	a	***	*Gammaproteobacteria*
**ASV_046**	0 ± 0	b	0.05 ± 0.08	b	0.14 ± 0.08	b	1.07 ± 1.01	a	***	*Gammaproteobacteria*
ASV_200	0 ± 0		0 ± 0		0 ± 0		0.37 ± 0.81			*Gammaproteobacteria*
ASV_236	0 ± 0		0 ± 0		0.02 ± 0.04		0.18 ± 0.25			*Gammaproteobacteria*

Table S4. continued.

Close taxon[a] / ASV ID[b]	Sampling month[c] June	July	August	September	Sig.[d]	Note
Pseudomonas (14 ASVs)						
**ASV_020**	1.39 ± 1.16 ab	2.15 ± 2.05 a	0.18 ± 0.10 c	0.36 ± 0.28 bc	**	*Gammaproteobacteria*
ASV_072	1.23 ± 2.21	0.16 ± 0.23	0.14 ± 0.19	0.25 ± 0.37		*Gammaproteobacteria*
ASV_101	0.62 ± 1.08	0 ± 0	0.16 ± 0.36	0.03 ± 0.07		*Gammaproteobacteria*
ASV_160	0.25 ± 0.41	0.34 ± 0.31	0.06 ± 0.10	0 ± 0		*Gammaproteobacteria*
ASV_307	0.23 ± 0.33	0 ± 0	0.02 ± 0.04	0.03 ± 0.07		*Gammaproteobacteria*
ASV_245	0.22 ± 0.54	0.19 ± 0.22	0 ± 0	0 ± 0		*Gammaproteobacteria*
ASV_238	0.11 ± 0.26	0.17 ± 0.36	0.04 ± 0.10	0 ± 0		*Gammaproteobacteria* (*P. umsongensis*)
ASV_243	0.04 ± 0.07	0.18 ± 0.36	0.01 ± 0.01	0 ± 0		*Gammaproteobacteria* (*P. viridiflava*)
ASV_302	0 ± 0	0.19 ± 0.20	0 ± 0	0.06 ± 0.14		*Gammaproteobacteria*
Unclassfied *Sinobacteraceae* (26 ASVs)						
**ASV_024**	0.20 ± 0.18 b	0.48 ± 0.20 b	0.40 ± 0.09 b	1.91 ± 0.78 a	***	*Gammaproteobacteria*
ASV_106	0.04 ± 0.09 b	0.14 ± 0.11 ab	0.23 ± 0.11 a	0.30 ± 0.13 a	***	*Gammaproteobacteria*
ASV_176	0.03 ± 0.07 b	0.09 ± 0.11 b	0.00 ± 0.01 b	0.26 ± 0.11 a	***	*Gammaproteobacteria*
ASV_265	0 ± 0 b	0 ± 0 b	0.05 ± 0.06 ab	0.11 ± 0.11 a	**	*Gammaproteobacteria*
ASV_320	0 ± 0 b	0 ± 0 b	0.01 ± 0.02 b	0.10 ± 0.06 a	***	*Gammaproteobacteria*
Unclassfied *Steroidobacter* in *Sinobacteraceae* (13 ASVs)						
**ASV_026**	0.25 ± 0.08 b	0.48 ± 0.20 b	0.63 ± 0.41 b	1.78 ± 0.34 a	***	*Gammaproteobacteria*
**ASV_011**	0.03 ± 0.04 c	0.11 ± 0.09 c	0.72 ± 0.26 b	3.19 ± 1.17 a	***	*Gammaproteobacteria*
ASV_082	0.02 ± 0.04 c	0.09 ± 0.08 bc	0.17 ± 0.08 b	0.66 ± 0.25 a	***	*Gammaproteobacteria*
Unclassified *Dokdonella* (12 ASVs)						
**ASV_027**	0.15 ± 0.09 c	0.53 ± 0.11 b	1.02 ± 0.19 a	0.93 ± 0.36 ab	***	*Gammaproteobacteria*
ASV_138	0.07 ± 0.07	0.10 ± 0.09	0.21 ± 0.06	0.11 ± 0.08		*Gammaproteobacteria*
ASV_186	0.04 ± 0.07	0.07 ± 0.11	0.07 ± 0.08	0.19 ± 0.17		*Gammaproteobacteria*
ASV_136	0.03 ± 0.05 b	0.14 ± 0.07 a	0.15 ± 0.12 ab	0.16 ± 0.10 a	*	*Gammaproteobacteria*
Unclassified *Rhodanobacter* (6 ASVs)						
**ASV_019**	1.88 ± 1.03 a	1.16 ± 0.27 a	0.92 ± 0.35 ab	0.34 ± 0.31 b	***	*Gammaproteobacteria*
ASV_107	0.44 ± 0.30	0.33 ± 0.22	0.14 ± 0.12	0.13 ± 0.12		*Gammaproteobacteria*
ASV_178	0.11 ± 0.26	0.21 ± 0.30	0.12 ± 0.12	0 ± 0		*Gammaproteobacteria*
ASV_053	0 ± 0 b	0.48 ± 0.54 ab	0.72 ± 0.80 a	0.42 ± 0.51 ab	*	*Gammaproteobacteria*

[a]Numbers of ASV belonging to each taxon are shown in parentheses. ASVs with less than 0.1% of relative abundance in all sampling months are not shown.

[b]ASVs with 0.1% or more than 0.1% of relative abundance in any one of sampling months are shown. Relative abundance was caluculated based on 5491 reads per sample. ASVs listed in Table 4 are indicated in bold font.

[c]Results of average ± S.D. (n=6) are shown. The same letter indicate no statistical significance among the months and months with high, low and moderate abundances are highlited with dark gray, white and light gray backgrounds, respectively.

[d]*, **, and *** indicate the statistical significance among sampling months by one-way ANOVA at $P < 0.05$, $P < 0.01$, and $P < 0.001$, respectively.

Table S5. Results of Blast analyses with representative seqences of ASVs showing statistical differences for their relative abundances among the sampling months

ASV[a]	Length (base)	Closest known species (Acession No.)	Score (bit)	Expect	Identity (%)
ASV_001 (*Streptomyces*)[b]	253	*Streptomyces aurantiacus* (OM909107)	457	3.E-124	100
	253	*Streptomyces cacaoi* (MZ165333)	457	3.E-124	100
	253	*Streptomyces cacaoi* (ON217557)	457	3.E-124	100
	253	*Streptomyces davaonensis* (OK067768)	457	3.E-124	100
	253	*Streptomyces dioscori* (OM971584)	457	3.E-124	100
	253	*Streptomyces ederensis* (MW363055)	457	3.E-124	100
	253	*Streptomyces fagopyri* (MZ254825)	457	3.E-124	100
	253	*Streptomyces lacrimifluminis* (MW642153)	457	3.E-124	100
	253	*Streptomyces neopeptinius* (OK135801)	457	3.E-124	100
	253	*Streptomyces ossamyceticus* (MZ254802)	457	3.E-124	100
	253	*Streptomyces platensis* (OM818541)	457	3.E-124	100
	253	*Streptomyces rishiriensis* (MZ413773)	457	3.E-124	100
	253	*Streptomyces scabiei* (OM971414)	457	3.E-124	100
	253	*Streptomyces tauricus* (OM541329)	457	3.E-124	100
	253	*Streptomyces turgidiscabies* (OL691265)	457	3.E-124	100
	253	*Streptomyces umbrinus* (MZ292113)	457	3.E-124	100
ASV_002 (*Chitinophagaceae*)	253	*Niastella populi* (AB682649)	430	4.E-116	97.6
ASV_003 (*Caulobacter henricii*)	253	*Caulobacter henricii* (MH929866)	457	3.E-124	100
	253	*Caulobacter mirabilis* (MN684273)	457	3.E-124	100
	253	*Caulobacter rhizosphaerae* (MT373603)	457	3.E-124	100
	253	*Caulobacter soli* (NR_174334)	457	3.E-124	100
ASV_005 (*Chitinophaga arvensicola*)	253	*Chitinophaga ginsengisegetis* (MT856245)	457	3.E-124	100
	253	*Luteifibra arvensicola* (AM237315)	457	3.E-124	100
ASV_006 (*Rhizobium*)	253	*Rhizobium leguminosarum* (ON201512)	457	3.E-124	100
	253	*Rhizobium multihospitium* (OM909424)	457	3.E-124	100
	253	*Rhizobium sophorae* (OM736176)	457	3.E-124	100
ASV_007 (*Sphingomonas*)	253	*Sphingomonas asaccharolytica* (MN181194)	457	3.E-124	100
	253	*Sphingomonas mali* (JN592470)	457	3.E-124	100
	253	*Sphingomonas melonis* (MN181188)	457	3.E-124	100
	253	*Sphingomonas pruni* (MK192021)	457	3.E-124	100
ASV_009 (*Agrobacterium*)	253	*Agrobacterium cavarae* (OM971167)	457	3.E-124	100
	253	*Agrobacterium larrymoorei* (MT367829)	457	3.E-124	100
	253	*Agrobacterium rosae* (MZ389115)	457	3.E-124	100
	253	*Agrobacterium rubi* (LC667774)	457	3.E-124	100
	253	*Agrobacterium salinitolerans* (MZ203655)	457	3.E-124	100
	253	*Agrobacterium tumefaciens* (MZ768713)	457	3.E-124	100
	253	*Agrobacterium vaccinii* (MT46040)	457	3.E-124	100
	253	*Neorhizobium alkalisoli* (MT634586)	457	3.E-124	100
	253	*Neorhizobium galegae* (CP090095)	457	3.E-124	100
	253	*Neorhizobium tomejilense* (MW748050)	457	3.E-124	100
	253	*Neorhizobium vignae* (MW748048)	457	3.E-124	100
	253	*Rhizobium panacihumi* (OM971096)	457	3.E-124	100
	253	*Rhizobium skierniewicense* (MN826327)	457	3.E-124	100

Table S5. Continued.

ASV[a]	Length (base)	Closest known species (Acession No.)	Score (bit)	Expect	Identity (%)
ASV_010 (*Novosphingobium*)	253	*Novosphingobium naphthalenivorans* (AB649005)	448	2.E-121	99.2
ASV_011 (*Steroidobacter*)	253	*Steroidobacter flavus* (KU195414)	416	9.E-112	96.4
	253	*Steroidobacter agariperforans* (MW186159)	416	9.E-112	96.4
ASV_012 (*Kribbella*)	253	*Kribbella albertanoniae* (MT415193)	457	3.E-124	100
	253	*Kribbella antibiotica* (NR_029048)	457	3.E-124	100
	253	*Kribbella endophytica* (KX502948)	457	3.E-124	100
	253	*Kribbella flavida* (KF620272)	457	3.E-124	100
	253	*Kribbella italica* (KJ875927)	457	3.E-124	100
	253	*Kribbella karoonensis* (NR_043327)	457	3.E-124	100
	253	*Kribbella pittospori* (MT239507)	457	3.E-124	100
	253	*Kribbella swartbergensis* (KP052783)	457	3.E-124	100
	253	*Kribbella turkmenica* (MG770857)	457	3.E-124	100
	253	*Nocardioides fulvus* (AJ871312)	457	3.E-124	100
ASV_013 (*Novosphingobium*)	253	*Novosphingobium naphthalenivorans* (AB649005)	448	2.E-121	99.2
ASV_014 (*Pedobacter*)	253	*Pedobacter cryoconitis* (KC788066)	457	3.E-124	100
	253	*Pedobacter foliorum* (MT441864)	457	3.E-124	100
	253	*Pedobacter ginsengisoli* (MT758241)	457	3.E-124	100
	253	*Pedobacter humi* (NR_149285)	457	3.E-124	100
	253	*Pedobacter panaciterrae* (MT102295)	457	3.E-124	100
	253	*Pedobacter quisquiliarum* (MT804242)	457	3.E-124	100
ASV_015 (*Sphingobium*)	253	*Sphingobium aromaticiconvertens* (MF101093)	457	3.E-124	100
ASV_016 (*Sphingomonas azotifigens*)	253	*Mesorhizobium plurifarium* (KJ609581)	457	3.E-124	100
	253	*Sphingomonas asaccharolytica* (MT634400)	457	3.E-124	100
	253	*Sphingomonas canadensis* (OM971160)	457	3.E-124	100
	253	*Sphingomonas kyeonggiensis* (MT634556)	457	3.E-124	100
	253	*Sphingomonas mali* (MN421334)	457	3.E-124	100
	253	*Sphingomonas mucosissima* (KM502884)	457	3.E-124	100
ASV_017 (*Amycolatopsis*)	253	*Amycolatopsis bullii* (KX502982)	457	3.E-124	100
	253	*Amycolatopsis eburnea* (MH598363)	457	3.E-124	100
	253	*Amycolatopsis kentuckyensis* (MT568584)	457	3.E-124	100
	253	*Amycolatopsis lexingtonensis* (MK968580)	457	3.E-124	100
	253	*Amycolatopsis mediterranei* (KF469352)	457	3.E-124	100
	253	*Amycolatopsis pretoriensis* (KX502981)	457	3.E-124	100
	253	*Amycolatopsis rifamycinica* (MT568585)	457	3.E-124	100
	253	*Amycolatopsis stemonae* (LC011703)	457	3.E-124	100
	253	*Amycolatopsis tolypomycina* (LC656369)	457	3.E-124	100
	253	*Amycolatopsis vancoresmycina* (HE966414)	457	3.E-124	100
	253	*Amycolatopsis vastitatis* (NR_164904)	457	3.E-124	100
ASV_019 (*Rhodanobacter*)	253	*Rhodanobacter hydrolyticus* (KY117469)	457	3.E-124	100
	253	*Rhodanobacter lindaniclasticus* (MH209632)	457	3.E-124	100
	253	*Rhodanobacter spathiphylli* (KU220865)	457	3.E-124	100
	253	*Rhodanobacter thiooxydans* (HG794332)	457	3.E-124	100
	253	*Rhodanobacter umsongensis* (NR_108435)	457	3.E-124	100
	253	*Rhodanobacter xiangquanii* (NR_132710)	457	3.E-124	100

Table S5. Continued.

ASV[a]	Length (base)	Closest known species (Acession No.)	Score (bit)	Expect	Identity (%)
ASV_020 (*Pseudomonas*)	253	*Pseudomonas arsenicoxydans* (MW854008)	457	3.E-124	100
	253	*Pseudomonas baetica* (OM533724)	457	3.E-124	100
	253	*Pseudomonas caricapapayae* (MW927165)	457	3.E-124	100
	253	*Pseudomonas coronafaciens* (MW927187)	457	3.E-124	100
	253	*Pseudomonas fluorescens* (ON072244)	457	3.E-124	100
	253	*Pseudomonas jessenii* (ON337522)	457	3.E-124	100
	253	*Pseudomonas kilonensis* (MZ642820)	457	3.E-124	100
	253	*Pseudomonas kitaguniensis* (MW927169)	457	3.E-124	100
	253	*Pseudomonas mandelii* (OK135850)	457	3.E-124	100
	253	*Pseudomonas migulae* (OM533710)	457	3.E-124	100
	253	*Pseudomonas mohnii* (OK393666)	457	3.E-124	100
	253	*Pseudomonas prosekii* (MZ045714)	457	3.E-124	100
	253	*Pseudomonas reinekei* (MW799926)	457	3.E-124	100
	253	*Pseudomonas silesiensis* (ON028726)	457	3.E-124	100
	253	*Pseudomonas synxantha* (ON056019)	457	3.E-124	100
	253	*Pseudomonas umsongensis* (MZ642912)	457	3.E-124	100
	253	*Pseudomonas umsongensis* (OM533715)	457	3.E-124	100
	253	*Pseudomonas vancouverensis* (MZ031292)	457	3.E-124	100
ASV_021 (*Chitinophaga*)	253	*Chitinophaga aurantiaca* (NR_164960)	434	3.E-117	98.0
	253	*Chitinophaga japonensis* (MF101181)	434	3.E-117	98.0
	253	*Chitinophaga ginsengihumi* (KY649382)	434	3.E-117	98.0
ASV_022 (*Oxalobacteraceae*)	253	*Herbaspirillum seropedicae* (HQ406764)	457	3.E-124	100
	253	*Herminiimonas fonticola* (MT992769)	457	3.E-124	100
	253	*Oxalicibacterium horti* (MH259951)	457	3.E-124	100
	253	*Oxalicibacterium solurbis* (NR_114175)	457	3.E-124	100
ASV_023 (*Novosphingobium*)	253	*Novosphingobium stygium* (MT825194)	448	2.E-121	99.2
	253	*Parablastomonas arctica* (KC759680)	448	2.E-121	99.2
ASV_024 (*Sinobacteraceae*)	253	*Acidibacter ferrireducens* (NR_126260)	398	2.E-106	94.9
ASV_025 (*Comamonadaceae*)	253	*Polaromonas aquatica* (MG763906)	457	3.E-124	100
	253	*Polaromonas cryoconiti* (KY302274)	457	3.E-124	100
	253	*Polaromonas eurypsychrophila* (MT373578)	457	3.E-124	100
	253	*Polaromonas glacialis* (MH482239)	457	3.E-124	100
	253	*Polaromonas hydrogenivorans* (KU179860)	457	3.E-124	100
	253	*Polaromonas jejuensis* (MT373588)	457	3.E-124	100
	253	*Variovorax boronicumulans* (MW186161)	457	3.E-124	100
ASV_026 (*Steroidobacter*)	253	*Steroidobacter flavus* (KU195414)	457	3.E-124	100
	253	*Steroidobacter agariperforans* (MW186159)	457	3.E-124	100
ASV_027 (*Dokdonella*)	253	*Dokdonella ginsengisoli* (KX255018)	457	3.E-124	100
	253	*Dokdonella soli* (NR_044554)	457	3.E-124	100

Table S5. Continued.

ASV[a]	Length (base)	Closest known species (Acession No.)	Score (bit)	Expect	Identity (%)
ASV_030 (*Janthinobacterium*)	253	*Janthinobacterium svalbardensis* (MT373677)	457	3.E-124	100
	253	*Massilia antarctica* (OM243916)	457	3.E-124	100
	253	*Massilia aquatica* (MN612042)	457	3.E-124	100
	253	*Massilia atriviolacea* (NR_171529)	457	3.E-124	100
	253	*Massilia aurea* (MN197561)	457	3.E-124	100
	253	*Massilia brevitalea* (MH667859)	457	3.E-124	100
	253	*Massilia eurypsychrophila* (MT373674)	457	3.E-124	100
	253	*Massilia frigida* (MN612049)	457	3.E-124	100
	253	*Massilia glaciei* (MH667841)	457	3.E-124	100
	253	*Massilia mucilaginosa* (MN612044)	457	3.E-124	100
	253	*Massilia niabensis* (MT373587)	457	3.E-124	100
	253	*Massilia puerhi* (MN014073)	457	3.E-124	100
	253	*Massilia rubra* (MN612022)	457	3.E-124	100
	253	*Massilia soli* (MT950109)	457	3.E-124	100
	253	*Massilia timonae* (MH127823)	457	3.E-124	100
	253	*Massilia violaceinigra* (MT373681)	457	3.E-124	100
ASV_032 (*Asticcacaulis biprosthecium*)	253	*Asticcacaulis benevestitus* (NR_042433)	425	2.E-114	97.2
	253	*Asticcacaulis biprosthecium* (NR_114729)	425	2.E-114	97.2
	253	*Asticcacaulis taihuensis* (OL773519)	425	2.E-114	97.2
	253	*Novosphingobium subterraneum* (HM032869)	425	2.E-114	97.2
ASV_034 (*Kutzneria*)	253	*Kutzneria albida* (NR_122053)	457	3.E-124	100
	253	*Kutzneria buriramensis* (NR_109430)	457	3.E-124	100
	253	*Kutzneria chonburiensis* (NR_145619)	457	3.E-124	100
	253	*Kutzneria kofuensis* (MT760419)	457	3.E-124	100
	253	*Kutzneria viridogrisea* (MT760436)	457	3.E-124	100
ASV_038 (*Kribbella*)	253	*Kribbella alba* (MT415180)	457	3.E-124	100
	253	*Kribbella catacumbae* (NR_042657)	457	3.E-124	100
	253	*Kribbella ginsengisoli* (KY432698)	457	3.E-124	100
	253	*Kribbella karoonensis* (FJ796408)	457	3.E-124	100
	253	*Kribbella koreensis* (FN178450)	457	3.E-124	100
	253	*Kribbella qitaiheensis* (NR_148308)	457	3.E-124	100
	253	*Kribbella sandramycini* (MT072122)	457	3.E-124	100

Table S5. Continued.

ASV[a]	Length (base)	Closest known species (Acession No.)	Score (bit)	Expect	Identity (%)
ASV_039 (*Streptomyces*)	253	*Streptomyces aureocirculatus* (OM534621)	457	3.E-124	100
	253	*Streptomyces avermitilis* (OL773534)	457	3.E-124	100
	253	*Streptomyces bobili* (OM478626)	457	3.E-124	100
	253	*Streptomyces bungoensis* (MZ703139)	457	3.E-124	100
	253	*Streptomyces bungoensis* (OM019293)	457	3.E-124	100
	253	*Streptomyces caeruleatus* (OM478629)	457	3.E-124	100
	253	*Streptomyces cellostaticus* (OK513247)	457	3.E-124	100
	253	*Streptomyces chartreusis* (OM019273)	457	3.E-124	100
	253	*Streptomyces cyaneochromogenes* (OL468465)	457	3.E-124	100
	253	*Streptomyces cyaneus* (OM478628)	457	3.E-124	100
	253	*Streptomyces cyslabdanicus* (OL468320)	457	3.E-124	100
	253	*Streptomyces fodineus* (OM019279)	457	3.E-124	100
	253	*Streptomyces galilaeus* (OM909121)	457	3.E-124	100
	253	*Streptomyces griseoruber* (OK148892)	457	3.E-124	100
	253	*Streptomyces lasalocidi* (OK083686)	457	3.E-124	100
	253	*Streptomyces lincolnensis* (OL614842)	457	3.E-124	100
	253	*Streptomyces neopeptinius* (OM909118)	457	3.E-124	100
	253	*Streptomyces olivaceoviridis* (ON364563)	457	3.E-124	100
	253	*Streptomyces olivochromogenes* (ON361562)	457	3.E-124	100
	253	*Streptomyces osmaniensis* (OM971471)	457	3.E-124	100
	253	*Streptomyces plumbiresistens* (OM478614)	457	3.E-124	100
	253	*Streptomyces prunicolor* (ON231552)	457	3.E-124	100
	253	*Streptomyces qaidamensis* (MZ854154)	457	3.E-124	100
	253	*Streptomyces shaanxiensis* (MZ854153)	457	3.E-124	100
ASV_040 (*Comamonadaceae*)	253	*Rhizobacter bergeniae* (MN598643)	457	3.E-124	100
	253	*Rhizobacter gummiphilus* (MH174432)	457	3.E-124	100
	253	*Methylibium sp.* (KM187564)	457	3.E-124	100
ASV_046 (*Gammaproteobacteria*)	253	*Thiohalobacter thiocyanaticus* (NR_116699)	389	1.E-103	94.1
ASV_054 (*Gammaproteobacteria*)	253	*Thiohalobacter thiocyanaticus* (AP018052)	403	6.E-108	95.3
ASV_064 (*Streptomycetaceae*)	253	*Streptomyces achromogenes* (OM200076)	457	3.E-124	100
	253	*Streptomyces asoensis* (CP049838)	457	3.E-124	100
	253	*Streptomyces dioscori* (MT856259)	457	3.E-124	100
	253	*Streptomyces ederensis* (MT322154)	457	3.E-124	100
	253	*Streptomyces galbus* (MN314487)	457	3.E-124	100
	253	*Streptomyces phaeochromogenes* (MN421416)	457	3.E-124	100
	253	*Streptomyces torulosus* (MT322181)	457	3.E-124	100
	253	*Streptomyces umbrinus* (MN684267)	457	3.E-124	100
	253	*Streptomyces phaeofaciens* (MK431531)	457	3.E-124	100
	253	*Streptomyces montanisoli* (MW228048)	457	3.E-124	100
	253	*Streptomyces cinereoruber* (MT760637)	457	3.E-124	100
ASV_079 (*Myxococcales*)	253	*Aetherobacter rufus* (NR_148645)	379	8.00E-101	93.7
	253	*Chondromyces lanuginosus* (FJ176774)	379	8.00E-101	93.7

[a]ASVs showing statistical differences for their relative abundances among the sampling months

[b]Closest taxon to a representative sequence of an ASV is shown in parentheses.

Table S6. Changes of relative abundances (%) during the growth period from June to September for sugar beet lateral root-associated bacterial groups which have been reported to have plant growth promoting or inhibiting effect for sugar beet in previous studies

Genus[a]	Sampling month[b] June		July		August		September		Sig.[c]	PGP activity[d]	References
Streptomyces	15.7 ± 5.7	a	8.3 ± 3.0	b	7.0 ± 1.4	b	3.4 ± 1.2	c	***	N	Okazaki et al., 2021
Pseudomonas	4.1 ± 2.3	a	3.4 ± 2.1	a	0.6 ± 0.4	b	0.8 ± 0.6	b	***	P	Kloepper et al., 1980
										P	Dunne et al., 1998
										P	Çakmakçi et al., 2001
										P	Zachow et al., 2008
Sphingobium	2.3 ± 1.0	a	1.3 ± 0.4	b	1.2 ± 0.6	b	0.5 ± 0.2	c	***	P	Okazaki et al., 2021
Sphingomonas	1.9 ± 0.6	c	3.8 ± 0.8	ab	4.4 ± 0.8	a	2.7 ± 1.3	bc	***	P	Okazaki et al., 2021
Sphingopyxis	1.4 ± 0.8	a	0.9 ± 0.5	a	0.9 ± 0.4	a	0.1 ± 0.1	b	***	P	Okazaki et al., 2021
Mesorhizobium	1.3 ± 0.2	a	1.6 ± 0.2	a	1.5 ± 0.4	a	0.7 ± 0.2	b	***	P	Okazaki et al., 2021
Asticcacaulis	1.1 ± 0.2	a	1.1 ± 0.4	a	0.8 ± 0.4	a	0.1 ± 0.2	b	***	P	Okazaki et al., 2021
Burkholderia	0.9 ± 0.8		1.0 ± 1.0		1.3 ± 0.9		0.9 ± 1.0			P	Çakmakçi et al., 2001
Flavobacterium	0.9 ± 0.5	a	1.1 ± 0.6	a	0.2 ± 0.1	b	0.4 ± 0.5	ab	**	P	Zachow et al., 2008
Lysobacter	0.8 ± 0.4		1.2 ± 0.7		1.0 ± 0.5		0.7 ± 0.3			P	Zachow et al., 2008
Paenibacillus	0.28 ± 0.26	a	0.07 ± 0.05	ab	0.049 ± 0.043	ab	0.01 ± 0.02	b	*	P	Çakmakçi et al., 2006
Stenotrophomona	0.23 ± 0.31		0.05 ± 0.08		0.03 ± 0.02		0.06 ± 0.08			P	Dunne et al., 1998
Methylibium	0.2 ± 0.2	b	0.4 ± 0.1	ab	0.4 ± 0.1	ab	0.6 ± 0.3	a	*	N	Okazaki et al., 2021
Bacillus	0.12 ± 0.09	ab	0.22 ± 0.04	a	0.04 ± 0.04	b	0.11 ± 0.08	ab	**	P	Çakmakçi et al., 1999
										P	Zachow et al., 2008
										N	Okazaki et al., 2021
Polaromonas	0.05 ± 0.09	c	0.70 ± 0.23	a	0.35 ± 0.07	b	0.56 ± 0.17	ab	***	P	Okazaki et al., 2021
Chryseobacterium	0.05 ± 0.13		0.04 ± 0.04		0.012 ± 0.015		0.01 ± 0.01			P	Shi et al., 2009
Pantoea	0.01 ± 0.02		0		0		0			P	Zachow et al., 2008
										N	Okazaki et al., 2021
Nocardioides	0 ± 0		0.04 ± 0.05		0.02 ± 0.03		0.02 ± 0.04			P	Okazaki et al., 2021
Acinetobacter	0 ± 0		0 ± 0		0 ± 0		0.02 ± 0.02			P	Shi et al., 2009
Rhodobacter	0 ± 0		0 ± 0		0 ± 0		0.01 ± 0.01			P	Çakmakçi et al., 2006
Microbacterium	0 ± 0		0 ± 0		0.003 ± 0.007		0 ± 0			P	Zachow et al., 2008

[a]Families or closest taxa with 1% or more than 1% of relative abundance in any one of sampling months are shown. Relative abundance was caluculated based on 5491 reads per sample.

[b]Results of average ± S.D. (n=6) are shown. The same letter indicate no statistical significance among the months and months with high, low and moderate abundances are highlited with dark gray, white and light gray backgrounds, respectively.

[c]*, ** and *** indicate the statistical significance among months by one-way ANOVA at $P < 0.05$, $P < 0.01$ and $P < 0.001$, respectively.

[d]N and P stand for the plant growth inhibiting (negative) and promoting (positive) activity, respectively.

Fig. S1

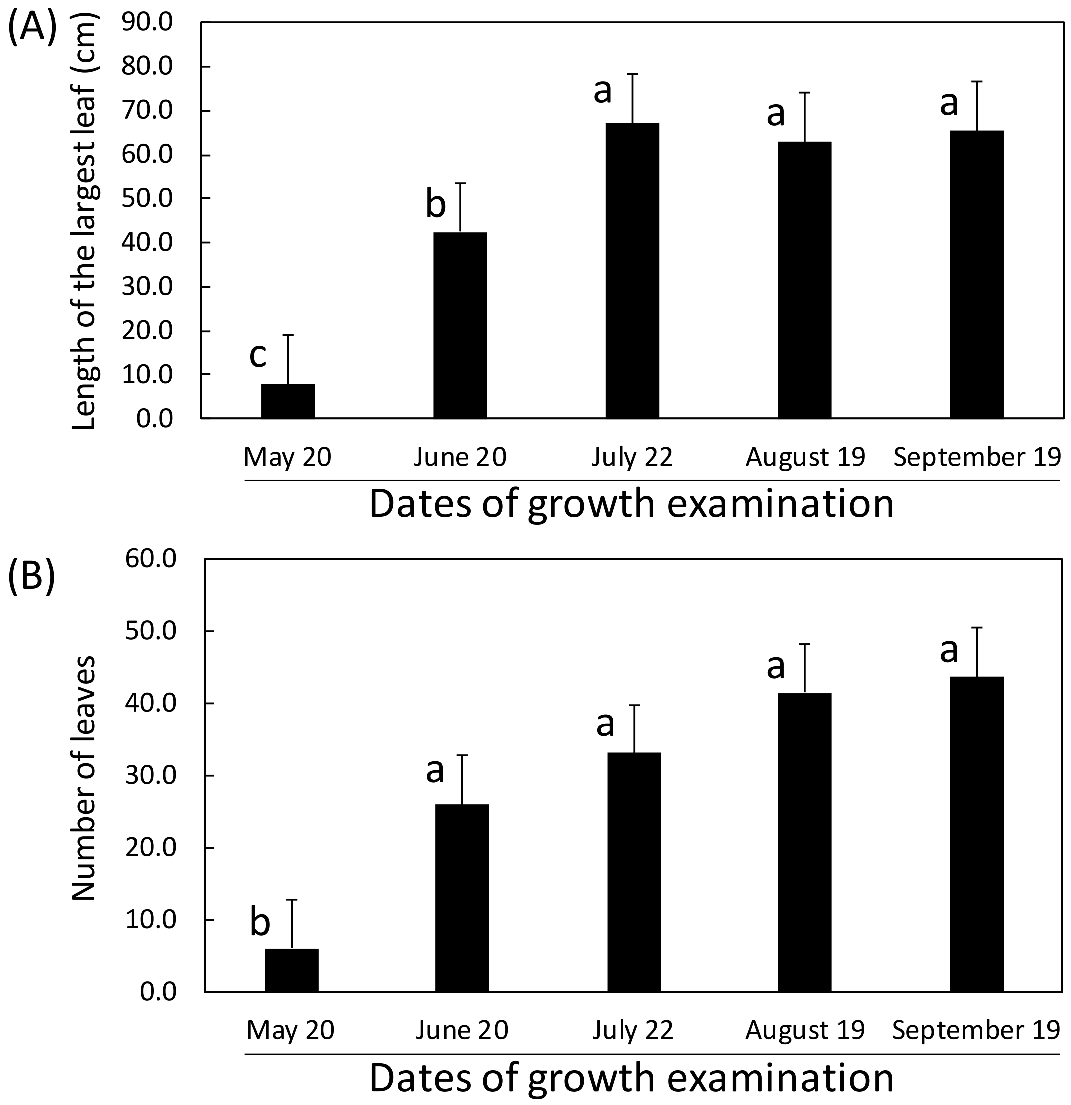

Fig. S1. The growth of above ground tissues of sugar beet cultivar "Rycka" planted in the andosol experimental field in the present study from May to September in 2014. Panels A and B indicate the averages of the leaf length of the largest leaf and the number of leaves per plant, respectively, in each of months examined. Error bar indicates standard error. The same letter indicates no statistical significance among the months. The results of one-way ANOVA indicated that there is a significant difference among the months examined at $P<0.001$ in both panels A and B.

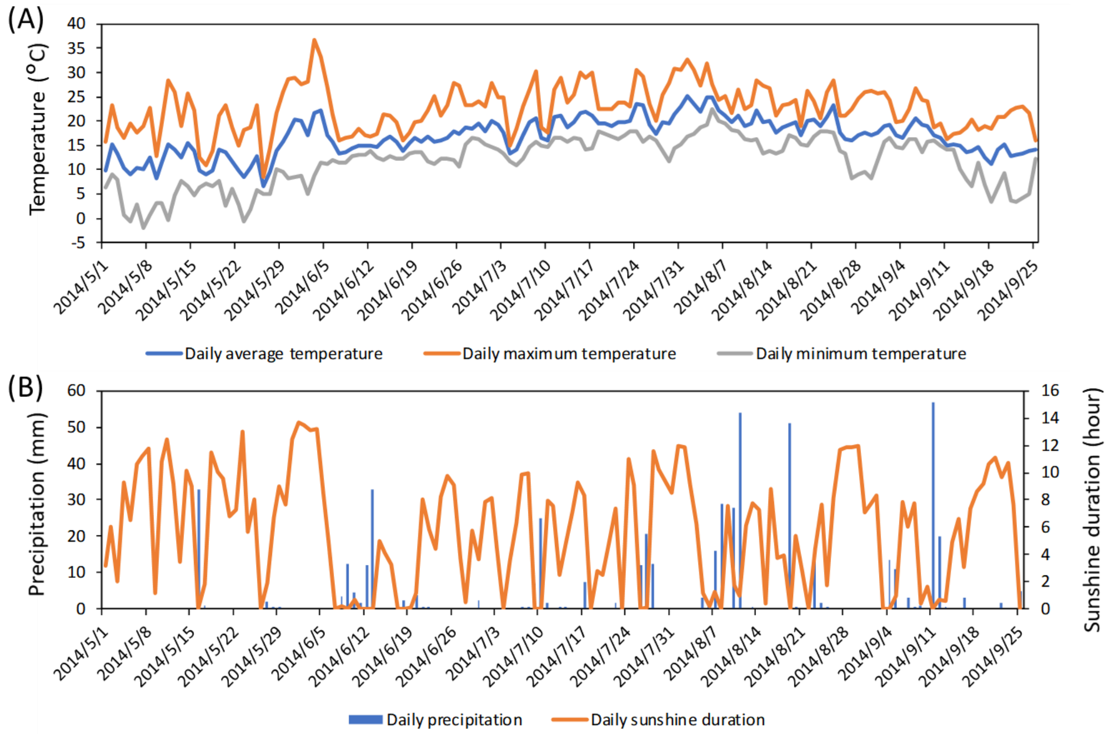

Fig. S2. The overview of weather condition for the andosol experimental field in the present study from May to September in 2014. Panel A indicates the daily average, maximum, and minimum temperatures. Panel B indicates the daily precipitation and sunshine duration time. Weather data were collected at the Meteorological Observation System of Memuro Upland Farming Research Division, Hokkaido Agricultural Research Center, NARO.

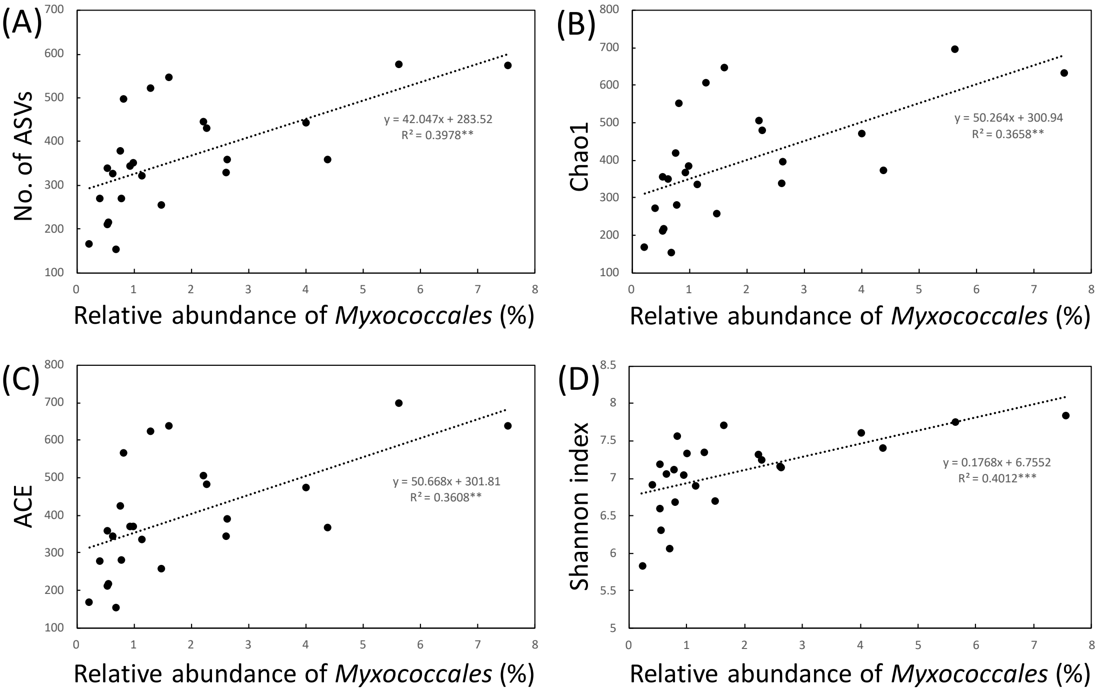

Fig. S3. Correlation plot of the relative abundance of *Myxococcales* and diversity indexes. ** and *** indicate a significant difference at P<0.01 and P<0.001, respectively.

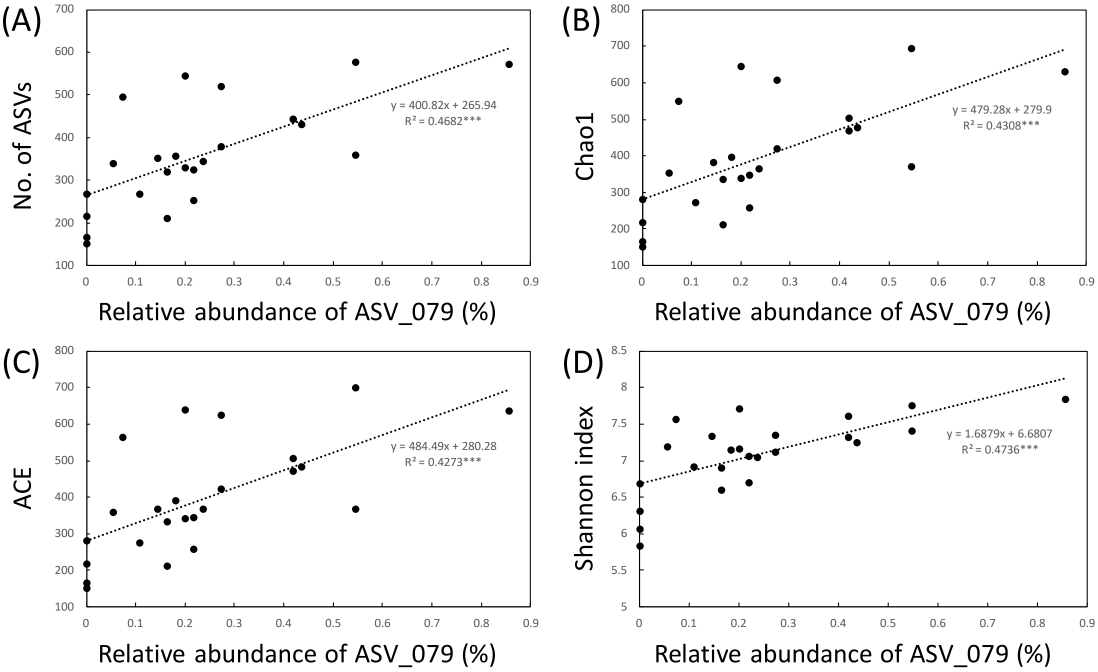

Fig. S4. Correlation plot of the relative abundance of ASV_79 (*Myxococcales*) and diversity indexes. *** indicates a significant difference at P<0.001.
